# Metabolic rewiring enables ammonium assimilation via a non‐canonical fumarate‐based pathway

**DOI:** 10.1111/1751-7915.14429

**Published:** 2024-03-14

**Authors:** Mohammad Saba Yousef Mardoukhi, Johanna Rapp, Iker Irisarri, Katrin Gunka, Hannes Link, Jan Marienhagen, Jan de Vries, Jörg Stülke, Fabian M. Commichau

**Affiliations:** ^1^ FG Molecular Microbiology, Institute for Biology University of Hohenheim Stuttgart Germany; ^2^ Interfaculty Institute for Microbiology and Infection Medicine Tübingen University of Tübingen Tübingen Germany; ^3^ Department of Applied Bioinformatics, Institute of Microbiology and Genetics, GZMB Georg‐August‐University Göttingen Göttingen Germany; ^4^ Campus Institute Data Science University of Göttingen Göttingen Germany; ^5^ Department of General Microbiology, Institute for Microbiology and Genetics, GZMB Georg‐August‐University Göttingen Göttingen Germany; ^6^ Institute of Bio‐ and Geosciences, IBG‐1: Biotechnology Forschungszentrum Jülich Jülich Germany; ^7^ Institut of Biotechnology RWTH Aachen University Aachen Germany

## Abstract

Glutamate serves as the major cellular amino group donor. In *Bacillus subtilis*, glutamate is synthesized by the combined action of the glutamine synthetase and the glutamate synthase (GOGAT). The glutamate dehydrogenases are devoted to glutamate degradation in vivo. To keep the cellular glutamate concentration high, the genes and the encoded enzymes involved in glutamate biosynthesis and degradation need to be tightly regulated depending on the available carbon and nitrogen sources. Serendipitously, we found that the inactivation of the *ansR* and *citG* genes encoding the repressor of the *ansAB* genes and the fumarase, respectively, enables the GOGAT‐deficient *B. subtilis* mutant to synthesize glutamate via a non‐canonical fumarate‐based ammonium assimilation pathway. We also show that the de‐repression of the *ansAB* genes is sufficient to restore aspartate prototrophy of an *aspB* aspartate transaminase mutant. Moreover, in the presence of arginine, *B. subtilis* mutants lacking fumarase activity show a growth defect that can be relieved by *aspB* overexpression, by reducing arginine uptake and by decreasing the metabolic flux through the TCA cycle.

## Graphical abstract

Glutamate is an abundant cellular metabolite that serves as a major donor of amino groups for the synthesis of nitrogen‐containing metabolites. Many bacteria can use either the glutamate dehydrogenase‐ or the glutamine synthetase/glutamate synthase‐dependent route for de novo synthesis of glutamate. Here, we show that the cellular requirement for glutamate in the soil bacterium *Bacillus subtilis* can be met by a non‐canonical fumarate‐based ammonium assimilation pathway.

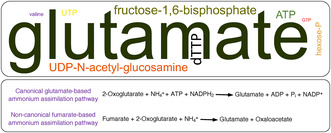

## INTRODUCTION

Glutamate is the major amino group donor in any living organism due to delivering 80–88% of the nitrogen for the synthesis of nitrogen‐containing molecules (Ikeda et al., 1996; Magasanik, 1996, 2003; Wohlheuter et al., 1973). Beside its role as a precursor for the synthesis of the glutamate family amino acids, such as glutamine, arginine, and proline, it is also directly incorporated into proteins. To a lesser extent but still important, glutamine also functions as an amino group donor for anabolic reactions (Wohlheuter et al., 1973). Therefore, it is not surprising that glutamate is the dominating metabolites in prokaryotic and eukaryotic cells (Bennett et al., 2009; Park et al., 2016). In the Gram‐positive model bacterium *Bacillus subtilis*, glutamate serves as an amino group donor in more than 30 transamination reactions (Oh et al., 2007).

Glutamate also serves as a counterion for potassium ions, which are the most abundant positively charged cellular ions (Epstein, 2003). The physiological importance of the link between glutamate and potassium was demonstrated in *E. coli* that responds to an increase in medium osmolarity by accumulating potassium ions and glutamate (McLaggan et al., 1994). Recent studies revealed that the link between glutamate and potassium homeostasis also exists in *B. subtilis* (Gundlach et al., 2018; Krüger et al., 2020, 2021). Moreover, glutamate itself may serve as an osmoprotectant in many archaea and bacteria (Csonka et al., 1994; Frank et al., 2021; Saum et al., 2006). In *B. subtilis*, glutamate is converted to proline that serves as a compatible solute to protect the cells under hyperosmotic conditions (Bremer & Krämer, 2019; Brill et al., 2011; Hoffmann et al., 2017; Stecker et al., 2022; Zaprasis et al., 2013, 2014). Thus, glutamate fulfils a key role in basic metabolism and the adaptation to the environmental osmolarity (Gunka & Commichau, 2012).

**FIGURE 1:**
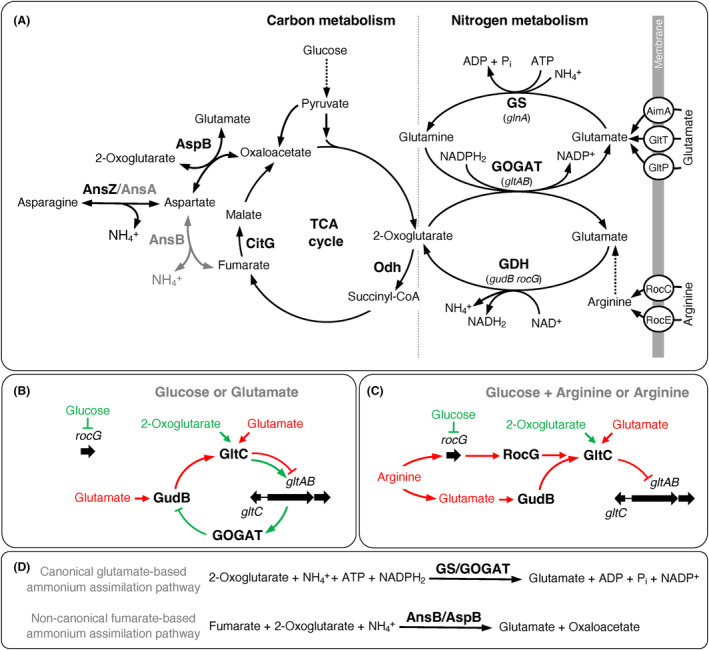
Links between carbon and nitrogen metabolism and regulation of glutamate biosynthesis in *Bacillus subtilis*. (A) Aspartate and glutamate metabolism and transporters for arginine and glutamate. AimA and GltT, glutamate transporters; RocC and RocE, arginine permeases; AnsA and AnsZ, asparaginases; AnsB, aspartase; AspB, aspartate transaminase; CitG, fumarase; Odh. 2‐oxoglutarate dehydrogenase enzyme complex; GDH, glutamate dehydrogenase encoded by *gudB* or *rocG*; GOGAT, glutamate synthase encoded by *gltAB*; GS, glutamine synthetase encoded by *glnA*; TCA, tricarboxylic acid. (B) Glucose‐ and glutamate‐dependent induction and repression, respectively, of the *gltAB* glutamate synthase genes. (C) Arginine‐dependent repression of the *gltAB* glutamate synthase genes. (D) Glutamate‐ and fumarate‐based ammonium assimilation pathways.

The enzymes catalysing the formation and degradation of glutamate link carbon to nitrogen metabolism (Figure 1A; Commichau et al., 2006; Sonenshein, 2007). Many organisms rely on a NADPH_2_‐dependent glutamate dehydrogenase (GDH) for producing glutamate from 2‐oxoglutarate and ammonium via reductive amination (Figure 1A; Hudson & Daniel, 1993). The GDH pathway was shown to be advantageous under energy limitation and at high external ammonium concentrations (Helling, 1994, 1998; Reizer, 2003). Alternatively, a NADPH_2_‐ or ferredoxin‐dependent glutamate synthase (GOGAT) can be used for converting 2‐oxoglutarate and glutamine into two molecules of glutamate (Figure 1A; Suzuki & Knaff, 2005). The ATP‐dependent glutamine synthetase (GS) converts ammonium and glutamate to glutamine that is required by the GOGAT (Kumada et al., 1993). In contrast to the GDH‐dependent ammonium assimilation, the GS‐GOGAT cycle is more efficient at low ammonium concentrations because the GS has a higher affinity for ammonium than the GDH (Helling, 1994, 1998; Reizer, 2003).

We are interested in glutamate metabolism of *B. subtilis* that possesses the GS‐GOGAT cycle and two GDHs (Belitsky & Sonenshein, 1998; Gunka & Commichau, 2012). The NAD^+^‐dependent GDHs RocG and GudB of *B. subtilis* are strictly devoted to glutamate degradation (Belitsky & Sonenshein, 1998; Commichau et al., 2008; Gunka et al., 2010). The *gudB* gene is constitutively expressed and the *rocG* gene is regulated by the available carbon and nitrogen sources (Gunka & Commichau, 2012). In the presence of glucose, *rocG* is repressed by the pleiotropic transcription factor CcpA (Belitsky et al., 2004; Choi & Saier Jr., 2005). Under these conditions, the transcription factor GltC activates the expression of the *gltAB* GOGAT genes (Figure 1B; Belitsky & Sonenshein, 2004; Bohannon & Sonenshein, 1989; Commichau et al., 2006; Wacker et al., 2003). Recently, it has been shown that the GOGAT GltAB binds to and inactivates the GDH GudB to prevent the degradation of glutamate (Figure 1B; Jayaraman et al., 2022). Thus, the GOGAT is a moonlighting protein required for glutamate synthesis and inactivation of GudB (Liu & Jeffery, 2020). Undomesticated strains of *B. subtilis* like NCIB 3610 can also use glutamate as the single source of carbon and nitrogen. Under these conditions, the catalytically active GDH GudB binds to and prevents GltC from activating the transcription of the *gltAB* genes (Figure 1B; Noda‐Garcia et al., 2017; Stannek et al., 2015). In the presence of arginine, which is converted via ornithine to glutamate, the DNA‐binding transcription factors AhrC and RocR, of which the latter is triggered by ornithine, activate the transcription of the *rocG* gene and the encoded GDH RocG (Belitsky & Sonenshein, 1998; Calogero et al., 1994; Gardan et al., 1995, 1997; Miller et al., 1997; Warneke et al., 2023). Like the GDH GudB, RocG can also inactivate the GltC protein (Figure 1C; Commichau, Herzberg, et al., 2007; Commichau, Wacker, et al., 2007; Stannek et al., 2015). The arginine‐dependent induction of the *rocG* gene dominates the glucose‐dependent negative regulation exerted by CcpA (Commichau, Herzberg, et al., 2007; Commichau, Wacker, et al., 2007; Stannek et al., 2015). Thus, the GDH RocG only comes into play when amino acids of the glutamate family (e.g., arginine) are available in the environment in high amounts (Stannek et al., 2015). The domesticated *B. subtilis* strain 168 harbours the cryptic *gudB* ^*CR*^ allele encoding an inactive GDH due to a tandem repeat in the open reading frame (Belitsky & Sonenshein, 1998; Gunka et al., 2012; Zeigler et al., 2008). However, the spontaneous decryptification of the *gudB* ^*CR*^ allele allow the bacteria to utilize amino acids of the glutamate family more efficient (Belitsky & Sonenshein, 1998; Gunka et al., 2012, 2013). *B. subtilis* can also take up glutamate from the environment via the glutamate transporters AimA and GltT (Krüger et al., 2021; Zaprasis et al., 2015).

Here, we show that the glutamate and aspartate auxotrophies of *B. subtilis* mutants lacking the GOGAT and aspartate transaminase AspB, respectively, are relieved by *ansR* and *citG* mutations that open an alternative entry point for ammonium via the reaction that is catalysed by the L‐aspartase AnsB. We also observed that the *B. subtilis citG* mutants lacking fumarase activity showed a growth defect in rich medium lacking glucose as an additional source of carbon. The growth defect of the *B. subtilis* mutant employing the non‐canonical fumarate‐based for pathway ammonium assimilation is probably due to the inability to utilize glutamate family amino acids as a carbon source.

## RESULTS

### Mutations in the *ansR* and *citG* genes relieve glutamate auxotrophy of a *gltAB* mutant

**FIGURE 2:**
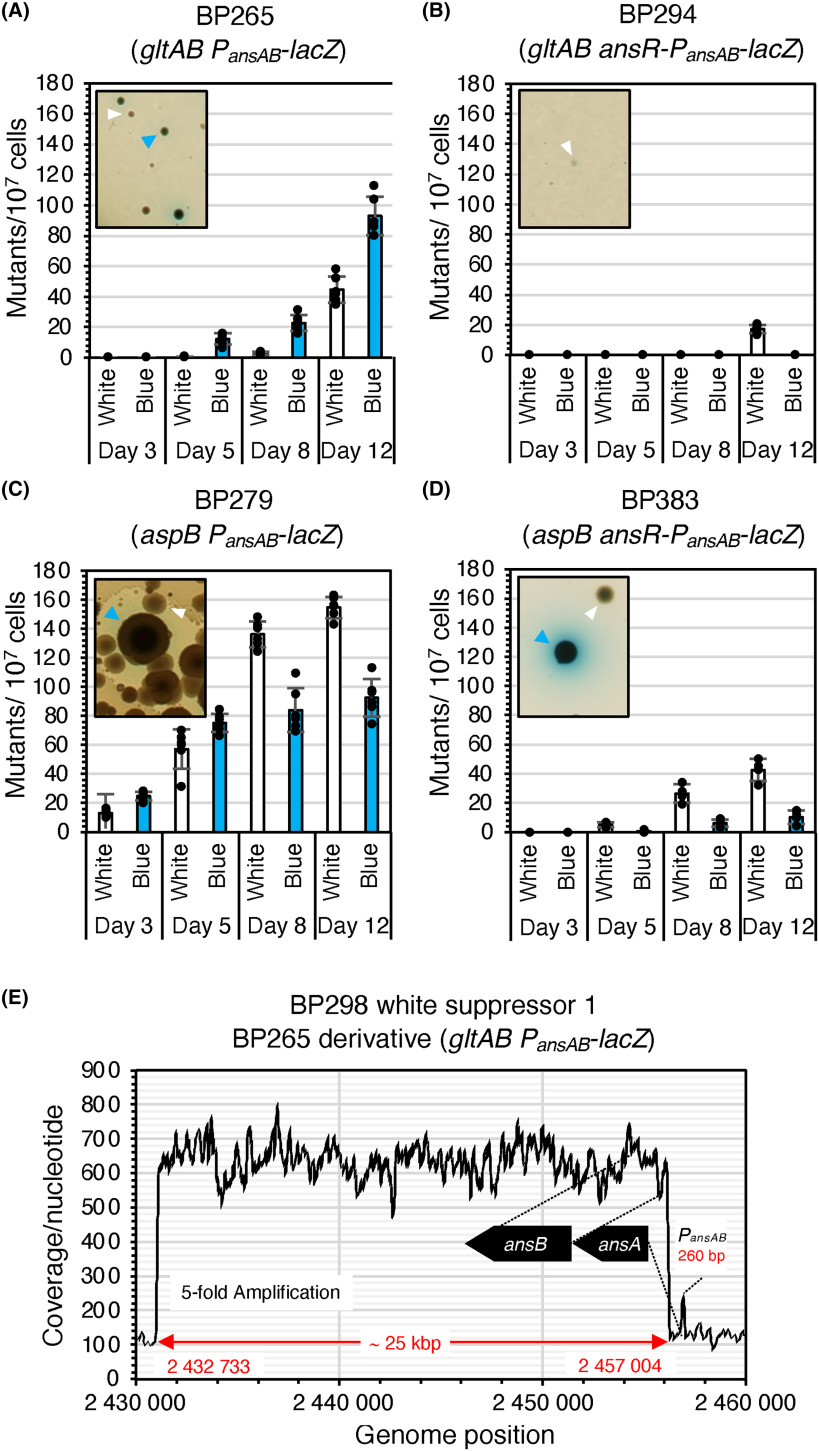
Genomic adaptation of *Bacillus subtilis aspB* and *gltAB* mutants abrogates aspartate and glutamate auxotrophy. (A–D) Formation of blue and white suppressor mutants (indicated by blue and white arrows) by the strains BP265 (*gltAB P*_*ansAB*_ *‐lacZ*), BP294 (*gltAB ansR‐P*_*ansAB*_ *‐lacZ*), BP279 (*aspB P*_*ansAB*_ *‐lacZ*) and BP383 (*aspB ansR‐P*_*ansAB*_ *‐lacZ*) harbouring a translational *ansAB* promoter‐*lacZ* fusion during growth under selection on CGXII plates. Strains BP294 and BP383 carry an additional *ansR* copy in the *amyE* locus. The activity of the *P*_*ansAB*_ promoter was visualized by adding the chromogenic substrate X‐Gal to the plates. Number or replicates in A–D and C, 6 and 4, respectively. Error bars represent standard deviation. (E) Read coverage along the chromosomal segment ranging from 2.430.000 to 24.600.000 bp. Based on the average coverage of the amplified region and of the remaining genome it can be inferred that five copies of the 25 kbp long region containing the entire *ansB* gene are present in the suppressor BP298.

Serendipitously, we observed that the *B. subtilis gltAB* mutant BP261 lacking the GOGAT formed single colonies on CGXII minimal medium plates during incubation for 5 days at room temperature (Figure 2A). CGXII medium that is commonly used for growth and maintenance of *Corynebacterium glutamicum*, contains glucose and ammonium/urea as sources of carbon and nitrogen, respectively (Keilhauer et al., 1993). Single colonies of two potential *gltAB* suppressor mutants designated as BP364 (M1) and BP365 (M2) as well as the parental strain BP261 (*gltAB*) as a control were propagated on CGXII plates. In contrast to the parental strain, the suppressor mutants grew after 48 h of incubation at 37°C (Figure S1A). Next, we verified the replacement of the *gltAB* gene by the tetracycline resistance gene in two suppressors by PCR (Figure S1B). To assess whether the suppressor mutations are genetically linked to the *gltAB* locus, we isolated the chromosomal DNAs of the two mutants and transformed the wild‐type strain SP1. Subsequent growth experiments revealed that the transformants were unable to grow on CGXII plates lacking glutamate. Thus, the mutation(s) in the mutants M1 and M2 are not genetically linked to the *gltAB* locus.

Next, we identified mutations in the mutants M1 and M2 by whole‐genome sequencing. As shown in Table 1, the sequencing analyses revealed that both mutants had acquired mutations affecting the *ansR* and *citG* genes. The *ansR* gene encodes the transcriptional repressor AnsR of the *ansAB* L‐asparaginase and L‐aspartase genes that are required for asparagine and aspartate degradation (Fisher & Wray, 2002; Sun & Setlow, 1991, 1993). The single nucleotide exchanges in the *ansR* alleles of M1 and M2 would cause the amino acid replacements C57R and L101P, respectively, in AnsR. The residues C57 and L101 are close to the helix‐turn‐helix motif and in the dimerization domain, respectively (Figure S1C). At this point, the amino acid replacements C57R and L101P may either enhance or decrease the function of AnsR. The *citG* gene codes for the fumarase CitG that catalyses the conversion of fumarate to malate in the tricarboxylic acid (TCA) cycle (Figure 1A; Feavers et al., 1988; Miles & Guest, 1985; Moir et al., 1984). While the mutant M1 does not synthesize the fumarase CitG because a 10.7 kbp‐long region (coordinates 3,381,391 to 3,392,113) including a big part of the *citG* gene was deleted, a single nucleotide insertion in the *citG* gene in mutant M2 would truncate the encoded protein (Table 1). Thus, two genomic alterations are sufficient to relieve glutamate auxotrophy of the *B. subtilis gltAB* mutant.

**TABLE 1:** Identified mutations in the *gltAB* and *aspB* suppressor mutants.

Strain	Mutant	Parental strain	Phenotype	Affected gene, mutations	Amino acid exchanges, effect on the protein
Mutations relieving glutamate auxotrophy of the strains BP261 (*gltAB*) and BP265 (*gltAB P*_*ansAB*_ *‐lacZ*)					
BP364	M1^a^	BP261 (*gltAB*)	Growth on CGXII	*ansR* T302C, 10.7 kbp deletion including *citG*	AnsR L101P, CitG not synthesized
BP365	M2^a^	BP261 (*gltAB*)	Growth on CGXII	*ansR* T169C, *citG* Δ62G	AnsR C57R, CitG ΔC43
BP281	S1B^b^	BP265 (*gltAB P*_*ansAB*_ *‐lacZ*)	Growth on CGXII, blue colonies	*ansR* + A36, *citG* + G917	AnsR ΔC11, CitG ΔC310
BP282	S2B^b^	BP265 (*gltAB P*_*ansAB*_ *‐lacZ*)	Growth on CGXII, blue colonies	*ansR* Δ2456934‐2457298	AnsR ΔC16
BP283	S3B^b^	BP265 (*gltAB P*_*ansAB*_ *‐lacZ*)	Growth on CGXII, blue colonies	*ansR* + A36, *citG* C1226A	AnsR ΔC11, CitG A409E
BP284	S4B^b^	BP265 (*gltAB P*_*ansAB*_ *‐lacZ*)	Growth on CGXII, blue colonies	*ansR* + A36	AnsR ΔC11
BP285	S5B^b^	BP265 (*gltAB P*_*ansAB*_ *‐lacZ*)	Growth on CGXII, blue colonies	*ansR* + A36	AnsR ΔC11
BP286	S6B^b^	BP265 (*gltAB P*_*ansAB*_ *‐lacZ*)	Growth on CGXII, blue colonies	*ansR* + A36	AnsR ΔC11
BP287	S7B^b^	BP265 (*gltAB P*_*ansAB*_ *‐lacZ*)	Growth on CGXII, blue colonies	*P*_*ansAB*_ (G‐10A), *citG* T876A	Enhanced *ansAB* expression, CitG D292E
BP288	S8B^b^	BP265 (*gltAB P*_*ansAB*_ *‐lacZ*)	Growth on CGXII, blue colonies	*ansR* + A36	AnsR ΔC11
BP289	S9B^b^	BP265 (*gltAB P*_*ansAB*_ *‐lacZ*)	Growth on CGXII, blue colonies	*ansR* + A36 *citG*, Δ3390211‐3390276	AnsR ΔC11, CitG not synthesized
BP290	S10B^b^	BP265 (*gltAB P*_*ansAB*_ *‐lacZ*)	Growth on CGXII, blue colonies	*ansR* + A36	AnsR ΔC11
BP298	S1W^a^	BP265 (*gltAB P*_*ansAB*_ *‐lacZ*)	Growth on CGXII, white colonies	*citG* A1033T, 25.2 kbp amplification including *ansB*	CitG I345F, increased AnsB synthesis
Mutations relieving aspartate and asparagine auxotrophy of the strain BP279 (*aspB P*_*ansAB*_ *‐lacZ*)					
BP366	S1B^b^	BP279 (*aspB P*_*ansAB*_ *‐lacZ*)	Growth on CGXII, blue colonies	*ansR* C171A	AnsR ΔC56
BP367	S2B^b^	BP279 (*aspB P*_*ansAB*_ *‐lacZ*)	Growth on CGXII, blue colonies	*ansR* G3A	AnsR M1I
BP368	S3B^b^	BP279 (*aspB P*_*ansAB*_ *‐lacZ*)	Growth on CGXII, blue colonies	*ansR* G94C	AnsR A32P
BP296	S1W^a^	BP279 (*aspB P*_*ansAB*_ *‐lacZ*)	Growth on CGXII, white colonies	*ansA* T43A, *citG* C686T	AnsA S15T, CitG A229V
BP297	S2W^a^	BP279 (*aspB P*_*ansAB*_ *‐lacZ*)	Growth on CGXII, white colonies	*ansA* T43A, *citG* C312G	AnsA S15T, CitG N104K

^a^ Mutations were identified by genome sequencing.

^b^ Mutations were identified by Sanger sequencing with primer pairs SM2/SM18 and SM3/SM4 for *P*_*ansAB*_ *‐ansR* and *citG*, respectively.

### A selection and screening system to distinguish *gltAB* suppressor mutants

To assess whether the inactivation of *ansR* is a prerequisite for restoring glutamate prototrophy of the *gltAB* mutant, we established a screening system. For this purpose, we introduced a translational *P*_*ansAB*_ *‐lacZ* into the *gltAB* mutant and propagated the strain BP265 (*gltAB P*_*ansAB*_ *‐lacZ*) on CGXII agar plates supplemented with the chromogenic substrate X‐Gal. Blue suppressor mutants would indicate the accumulation of loss‐of‐function mutations in the *ansR* gene. By contrast, white mutants indicate that the fusion is not active. The strain BP265 was cultivated overnight in 4 mL LB medium at 28°C. The cells were washed twice in 0.9% NaCl (w/v) solution and about 10^7^ cells were propagated on the plates. As shown in Figure 2A, several blue suppressor mutants appeared on the CGXII‐X‐Gal plate after incubation for 3 days at 37°C. Sanger sequencing of the *ansR* and *citG* genes in 10 isolated mutants revealed that all mutants had acquired mutations in the *ansR* gene or a mutation that likely affect translation (Figure S1D), and in four of the mutants, the *citG* gene was also affected (Table 1; Figure S1E,F). Thus, the de‐repression of the *ansAB* genes either by mutational inactivation of *ansR* or by mutations interfering with *ansR* expression is important for allowing the *gltAB* suppressors to synthesize glutamate via the AnsB/AspB‐dependent route (Figure 1A). Moreover, mutations in *citG* may contribute to redirect the metabolic flux to the aspartate branch.

Further incubation of the CGXII‐X‐Gal plates carrying the blue *gltAB* suppressor mutants for up to 8 days resulted in the emergence of additional white suppressors (Figure 2A). Illumina sequencing of one arbitrarily chosen white mutant BP298 revealed that the strain had amplified a 25.3 kbp‐long genomic segment causing a 5‐fold increased dosage of *ansB* and the surrounding genes (Figure 2E). Thus, the glutamate auxotrophy of the strain BP265 (*gltAB P*_*ansAB*_ *‐lacZ*) can also be relieved by enhancing the dosage of the *ansB* genes that is not expressed in the parental strain under these growth conditions. It is tempting to speculate that the blue mutants emerge earlier due to different frequencies of loss‐of‐function mutations in *ansR* and of selective gene amplifications of which the latter is a more costly process for the cell.

We also observed that the *gltAB* mutant BP294 (*gltAB ansR‐P*_*ansAB*_ *‐lacZ*) containing the native *ansR* gene and a second *ansR* copy in the *amyE* locus formed much less and only white mutants (Figure 2B). It remains elusive whether the white mutants carry mutations in *citG* or due to the amplification of a chromosomal segment carrying the *ansB* gene. However, *ansR* loss‐of‐function mutations are indeed crucial for relieving glutamate auxotrophy of a *gltAB* mutant. We also tested whether the strain BP280 (*gltAB ansAB*) was able to form suppressors on CGXII plates. However, the strain did not form suppressor mutants, indicating that no other route for glutamate de novo synthesis exists (data not shown).

To conclude, the reduced DNA‐binding activity or lack of AnsR cause the de‐repression of the *ansAB* genes, thereby allowing the GOGAT‐deficient bacteria to synthesize glutamate de novo from ammonium and glucose. It is tempting to speculate that the L‐aspartase AnsB synthesizes aspartate from ammonium and fumarate, and the concentration of the latter substrate probably increases due to *citG* mutations in some suppressors (Figure 1A,D). Moreover, we hypothesize that the aspartate transaminase AspB converts aspartate and 2OG to glutamate. Both, L‐aspartase and transaminase catalyse reversible reactions (Bennett et al., 2009; Viola, 2000).

### Inactivation of *ansR* relieves aspartate auxotrophy of an *aspB* mutant

*B. subtilis* synthesizes aspartate via the L‐aspartate transaminase AspB that converts oxaloacetate and glutamate to 2‐oxoglutarate and aspartate (Figure 1A). Previously, it has been shown that an *aspB* mutant is auxotrophic for aspartate or asparagine (Zhao et al., 2018). The study by Zhao et al. also revealed a slightly heterogenous colony morphology of a L‐aspartate transaminase‐deficient *B. subtilis* strain, indicating genetic instability of the mutant during growth on aspartate‐limited plates (figure 2C in Zhao et al., 2018). Moreover, given our observation that the reversible L‐aspartase AnsB likely converts ammonium and fumarate to aspartate, we hypothesized that the aspartate prototrophy of the *aspB* mutant could be restored by the inactivation of *ansR* (Figure 1A; Viola, 2000). To test this idea, we propagated the *aspB* mutant BP279 (*aspB P*_*ansAB*_ *‐lacZ*) on CGXII‐X‐Gal plates as described for the suppressor analysis with the strain BP265 (see above). Again, the translational *P*_*ansAB*_ *‐lacZ* fusion was used to facilitate the discrimination between different mutant classes. Blue and white colonies appeared on the plates after incubation for 3 days at 37°C (Figure 2C). Sanger sequencing of *ansR* in the three isolated blue *aspB* suppressors BP366, BP367, and BP368 revealed that the mutants had acquired loss‐of‐function mutations in the *ansR* gene that would inactivate the encoded repressor (Table 1; Figure S1C). Moreover, as observed with the *gltAB* mutant, the *aspB* mutant BP383 (*aspB ansR‐P*_*ansAB*_ *‐lacZ*) containing two *ansR* copies formed much less and only white mutations (Figure 2C). In contrast to the *gltAB* mutant strain BP265, the *aspB* mutant BP279 produced more suppressors, including significantly more that showed a white phenotype (Figure 2C). The observation that the *aspB* mutant forms quicker and more mutants than the *gltAB* mutant suggests that the selective pressure acting on the aspartate‐deficient strain is probably lower than that acting on the GOGAT‐deficient mutant. The genome sequencing analysis of the two isolated white *aspB* suppressors BP296 and BP297 revealed that both mutants had acquired mutations in the *citG* gene (Figure S1E,F; Table 1). Both mutants also carry a mutation in the *ansA* gene (Table 1). However, the effect of S15T replacement on AnsA activity needs to be analysed. The fact that the strain BP395 (*aspB ansAB*) did not form suppressors on CGXII plates suggests that the AspB reaction can only be bypassed by AnsB (data not shown). To conclude, the aspartate auxotrophy of the *aspB* mutant can be alleviated by mutations in *ansR* and *citG*.

### Characterization of mutants synthesizing glutamate and aspartate via AnsB

**FIGURE 3:**
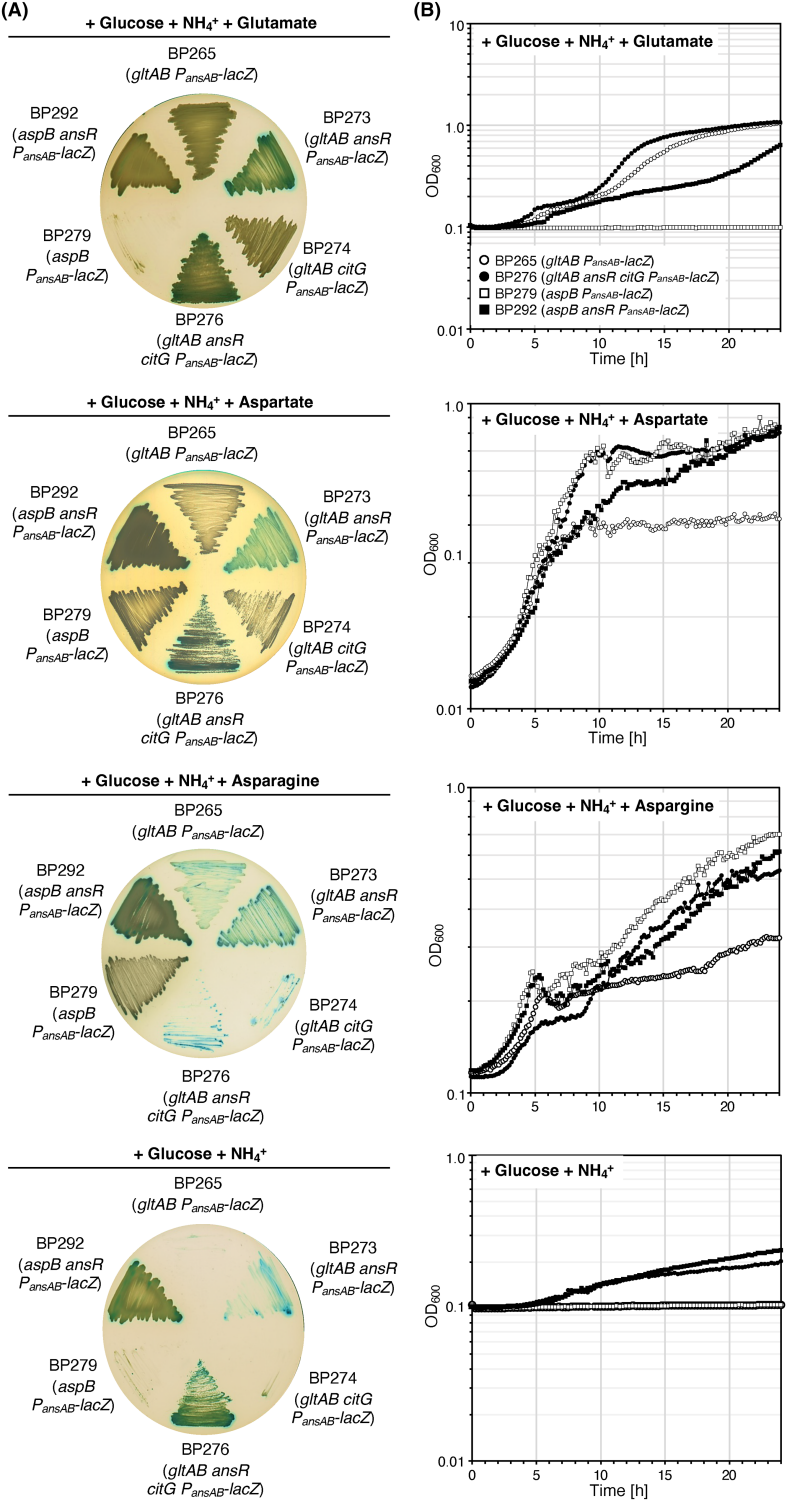
Characterization of *Bacillus subtilis* strains synthesizing aspartate and glutamate via metabolic bypasses. (A) The parental strains BP265 (*gltAB P*_*ansAB*_ *‐lacZ*) and BP279 (*aspB P*_*ansAB*_ *‐lacZ*) and the strains BP274 (*gltAB citG P*_*ansAB*_ *‐lacZ*), BP276 (*gltAB citG ansR P*_*ansAB*_ *‐lacZ*), and BP292 (*aspB ansR P*_*ansAB*_ *‐lacZ*) were propagated on CGXII plates containing glucose and ammonium as carbon and nitrogen sources, respectively. Aspartate, Asparagine, and glutamate were added to a final concentration of 0.5% (w/v). The activity of the *P*_*ansAB*_ promoter was visualized by adding the chromogenic substrate X‐Gal to the plates. The plates were incubated for 48 h at 37°C. (B) Growth of the strains indicated in (A). in CGXII liquid medium at 37°C. To remove the amino acids from the precultures, cells were washed twice in 0.9% (w/v) saline solution. Each experiment was carried out three times independently (*N* = 3).

To assess the growth behaviour of *gltAB* mutants synthesizing glutamate and aspartate via the L‐aspartase AnsB, we deleted the *ansR* and *citG* genes individually and in combination in the strain BP265 (*gltAB*). In the strain BP279 (*aspB*), we only tested the effect of *ansR* deletion. Next, we evaluated the growth of the strains BP273 (*gltAB ansR*), BP274 (*gltAB citG*), BP276 (*gltAB ansR citG*), and BP292 (*aspB ansR*) on CGXII agar in the absence and in the presence of either glutamate, aspartate, or asparagine. The parental strains BP265 (*gltAB*) and BP279 (*aspB*) served as controls. In liquid medium, the growth of the parental and the deletion strains was assessed. As shown in Figure 3A,B, except for the strain BP279 (*aspB*), which cannot convert glutamate to aspartate, the remaining strains grew on plates and in liquid medium supplemented with glutamate as an additional nitrogen source. Moreover, as expected, aspartate supported growth of all strains. By contrast, on agar plates supplemented with asparagine, the strains BP274 and BP276 lacking the *citG* fumarase gene showed a growth defect, which was not the case for strain BP276 in liquid medium (Figure 3A,B). Moreover, only the reconstituted strains BP276 (*gltAB ansR citG*) and BP292 (*aspB ansR*) showed the best growth on plates and in liquid medium containing ammonium as the single source of nitrogen. Thus, the auxotrophy for glutamate and aspartate of the *gltAB* and *aspB* mutants can be relieved by the inactivation of the *ansR citG* and *ansR* genes, respectively.

### The evolution of *gltAB* mutants depends on the availability of ammonium and urea

Standard minimal media such as C‐Glc and SM, commonly used for culturing *B. subtilis*, contain 25 and 15 mM ammonium sulphate, respectively, as a source of nitrogen. In contrast, CGXII medium contains 134 mM ammonium sulphate and 74 mM urea of which the latter can be degraded to ammonium and carbon dioxide. Therefore, we tested whether the availability of ammonium and urea would influence the emergence of *gltAB* suppressor mutants. For this purpose, we added the amounts of ammonium sulphate and urea that are present in CGXII medium either individually or in combination to C‐Glc medium and assessed the emergence of suppressor mutants derived from the strain BP265 (*gltAB P*_*ansAB*_ *‐lacZ*) as described above. After eight days of incubation at 37°C, no suppressor mutants appeared C‐Glc on plates without additional supplements (Figure S2). Few colonies appeared on C‐Glc‐urea plates and significantly more suppressors emerged in the presence of increased amounts of ammonium (Figure S2). As already observed with the CGXII medium, most mutants arose on C‐Glc‐urea medium with additional ammonium (Figure S2). From this, we conclude that the ammonium and urea present in CGXII medium promotes poor growth of the *gltAB* mutant, which is a prerequisite for the emergence suppressor mutants.

### Ammonium assimilation efficiency of the *gltAB ansR citG* and *aspB ansR* mutants

**FIGURE 4:**
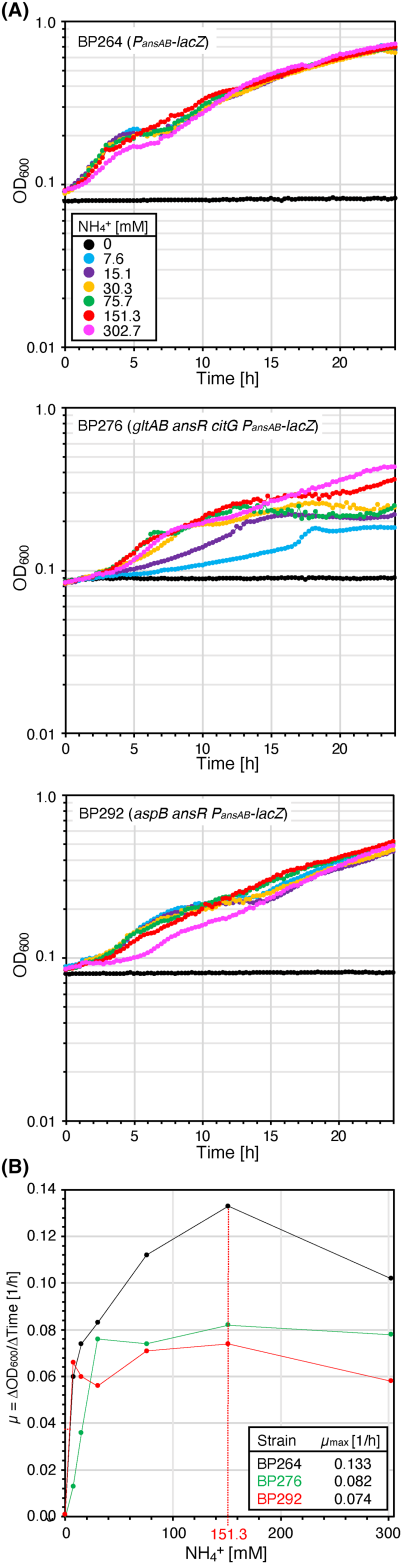
Ammonium dependency of *Bacillus subtilis* strains synthesizing aspartate and glutamate via metabolic bypasses. (A) The parental strain BP264 and the strains BP276 (*gltAB ansR citG*) and BP292 (*aspB ansR*) were cultivated at 37°C in SM medium supplemented with increasing amounts of ammonium. (B) Relationship between the ammonium concentration and the growth rate (μ). The maximum growth rate was reached at an ammonium concentration of 151.3 mM. Each experiment was carried out three times independently (*N* = 3).

To evaluate the efficiency of ammonium assimilation by the L‐aspartase AnsB in the strains BP276 (*gltAB ansR citG*) and BP292 (*aspB ansR*), we cultivated the bacteria in SM medium with increasing amounts of ammonium chloride (0–302.7 mM). The wild‐type strain BP264 served as a control. As expected, all strains did not grow in the absence of ammonium (Figure 4A). Albeit forming less biomass than the wild‐type, high ammonium concentrations also supported growth of the deletion strains BP276 (*gltAB ansR citG*) and BP292 (*aspB ansR*) (Figure 4A). The calculation of the growth rates revealed the assimilation of ammonium via AnsB is limited and less efficient than via the native GS‐GOGAT route (Figures 1A and 4B). Moreover, the strain BP276 showed the strongest dependency on ammonium. The lower ammonium dependency of the strain BP292 may be explained by the fact that in the background of the *aspB* aspartate transaminase mutation, the L‐aspartase‐catalysed ammonium assimilation reaction is only required for de novo synthesis of aspartate and asparagine (Figure 1A). In contrast, in the background of *gltAB* GOGAT mutant, the assimilation of ammonium via AnsB is needed for producing glutamate, which is the major amino group donor in the cell. To conclude, albeit less efficient, the alternative entry point for ammonium into central metabolism allows growth of the reconstituted mutants BP276 and BP292.

### Growth *gltAB ansR citG* and *aspB ansR* mutants under hyperosmotic conditions

**FIGURE 5:**
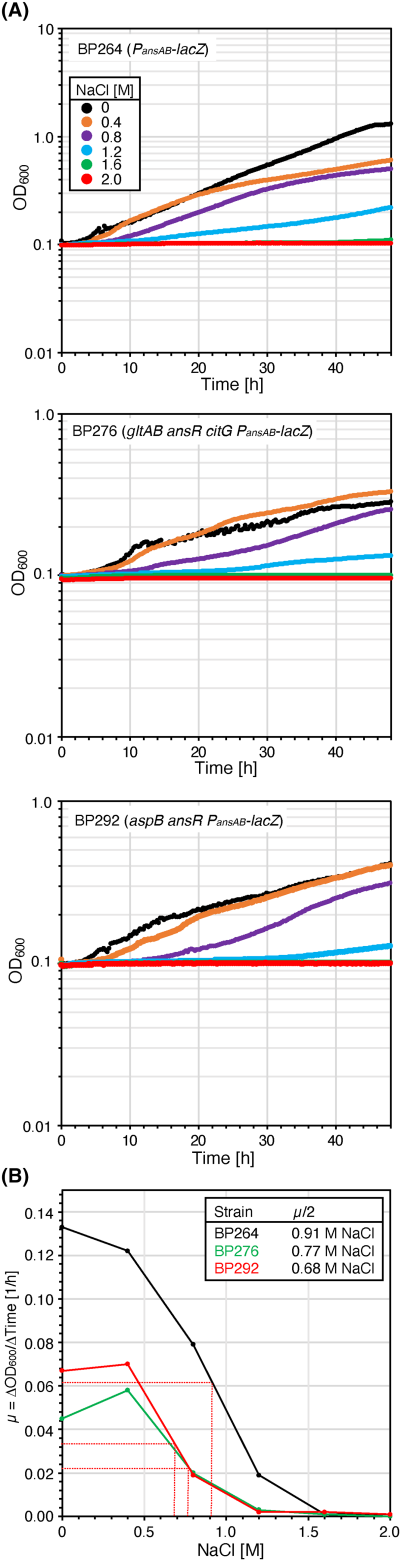
Effect of NaCl on growth of the *Bacillus subtilis* strains synthesizing aspartate and glutamate via metabolic bypasses. (A) The parental strain BP264 and the strains BP276 (*gltAB ansR citG*) and BP292 (*aspB ansR*) were cultivated at 37°C in SM medium supplemented with increasing amounts of NaCl. (B) Relationship between the NaCl concentration and the growth rate (μ). Each experiment was carried out three times independently (*N* = 3).

As described above, in addition to its role as the major amino group donor in the cell, glutamate serves as the precursor for the synthesis of L‐proline, which is as a compatible solute in *B. subtilis* under hyperosmotic growth conditions. Therefore, we assessed the ability of the deletion mutants BP276 (*gltAB ansR citG*) and BP292 (*aspB ansR*) to grow under hyperosmotic conditions. For this purpose, the strains including the wild‐type BP264 as a control were grown in SM medium supplemented with increasing amounts of sodium chloride (NaCl). As shown in Figure 5A,B, even though the growth of the deletion mutants was impaired at elevated NaCl concentrations, both strains synthesize significant amounts of glutamate to withstand an elevated environmental salinity.

**FIGURE 6:**
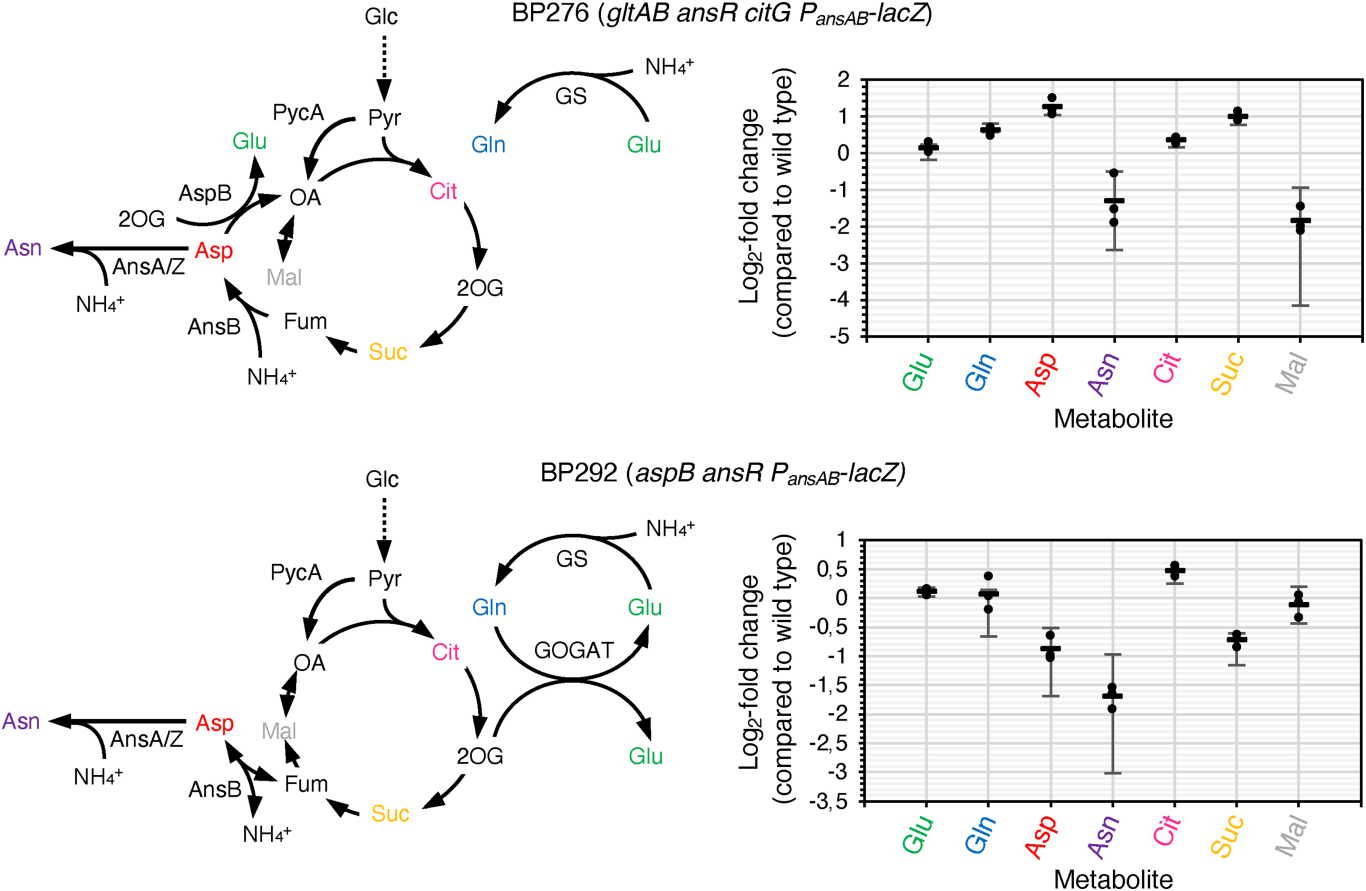
Concentrations of selected metabolites in *Bacillus subtilis* strains synthesizing aspartate and glutamate via metabolic bypasses. The parental strain BP264 and the strains BP276 (*gltAB ansR citG*) and BP292 (*aspB ansR*) were cultivated at 37°C in SM medium. The metabolites were identified by GC/MS and are shown as log_2_‐fold changes compared with the parental strain (see *Experimental procedures*). Mean value and standard deviation of three biological replicates are shown. Glc, glucose; Pyr, pyruvate; Cit, citrate; 2OG, 2‐oxoglutarate; Suc, succinate; Fum, fumarate; Mal, malate; OA, oxaloacetate; Asp, aspartate; Asn, asparagine; Glu, glutamate; Gln, glutamine. AnsA and AnsZ, asparaginases; AnsB, aspartase; AspB, aspartate transaminase; GOGAT, glutamate synthase; GS, glutamine synthetase. Each experiment was carried out three times independently (*N* = 3). Error bars represent standard deviation.

To determine the relative intracellular concentrations of glutamate, glutamine, aspartate, and asparagine as well as the TCA cycle intermediates citrate, succinate, and malate in the reconstituted mutants BP276 (*gltAB ansR citG*) and BP292 (*aspB ansR*), we performed metabolome analyses. As shown in Figure 6, both strains synthesized similar amounts of glutamate, glutamine, asparagine, and citrate. Moreover, the relative aspartate concentrations were increased and decreased in the strains BP276 and BP292, respectively (Figure 6). It is tempting to speculate that the aspartate concentration is elevated in the strain BP276 due to the deletion of *citG*. This idea is in line with the fact that the cellular concentration of malate is reduced in this strain (Figure 6). In contrast to strain BP276, the presence of the fumarase CitG probably explains a reduced cellular concentration of succinate in the strain BP292 (Figure 6). To conclude, despite significant changes in the metabolome of the reconstituted mutants, the metabolic network is robust enough to sustain growth of the bacteria using AnsB for ammonium assimilation.

### The active GDH enhances growth of the *gltAB ansR citG* mutant in rich medium

**FIGURE 7:**
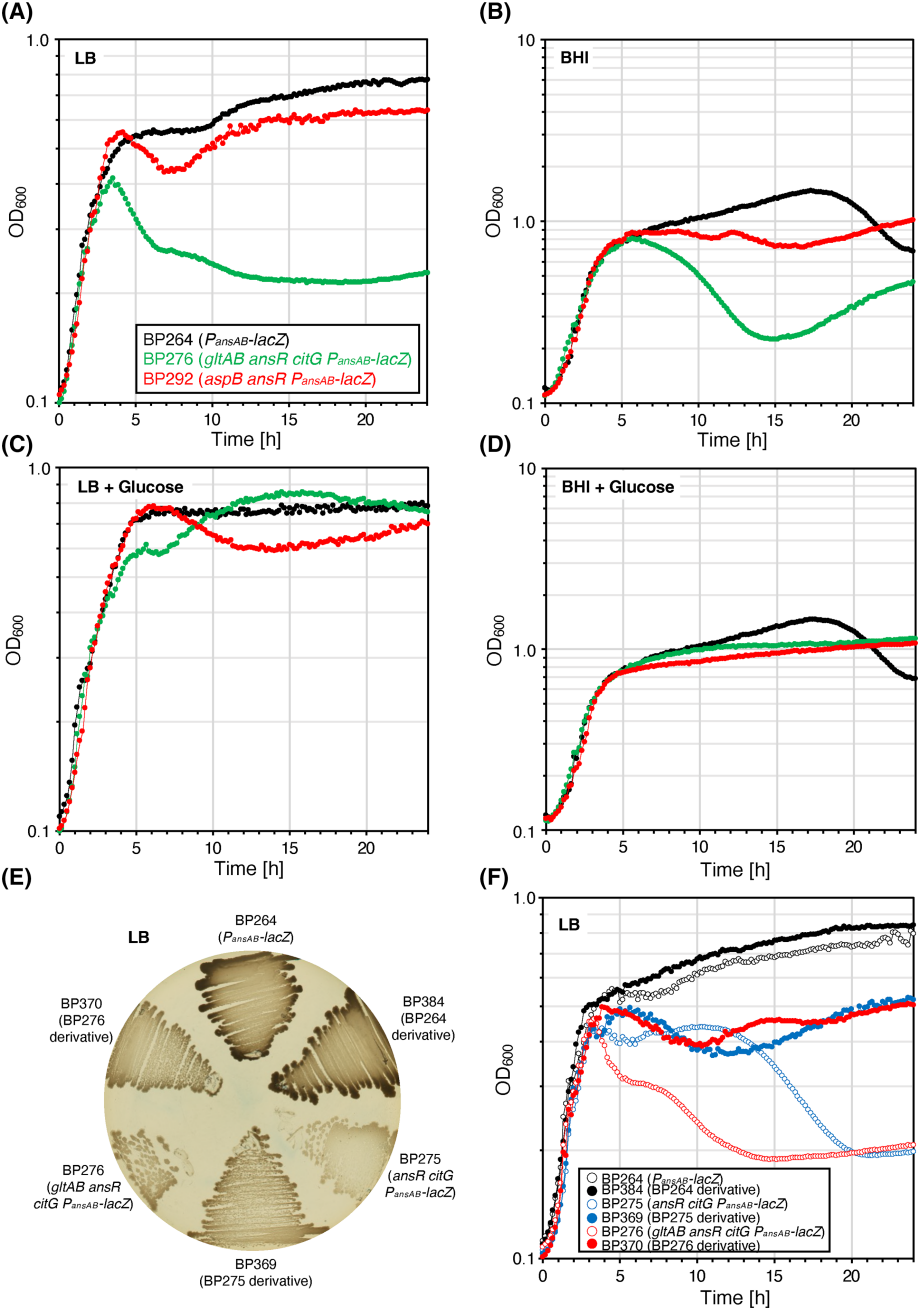
Adaptation of the *Bacillus subtilis* strains synthesizing aspartate and glutamate via metabolic bypasses to rich medium. The parental strain BP264 and the strains BP276 (*gltAB ansR citG*) and BP292 (*aspB ansR*) were cultivated at 37°C in LB and BHI medium without (A and B) and with glucose 0.5% (w/v) (C and D). (E) Agar plate showing the phenotypes of the strains BP264, BP276 (*gltAB ansR citG*), and BP292 (*aspB ansR*) and of the strains BP384, BP369, and BP370 that were evolved in LB liquid medium. The plate was incubated for 24 h at 37°C. (F) Growth of the strains BP264, BP276 (*gltAB ansR citG*), BP292 (*aspB ansR*), BP384 (LB evolved derivative of BP264), BP369 (LB evolved derivative of BP275), and BP370 (LB evolved derivative of BP276) in LB liquid medium at 37°C. Each experiment was carried out three times independently (*N* = 3).

During the construction of the strains BP275 (*ansR citG*) and BP276 (*gltAB ansR citG*) we observed that the bacteria lacking CitG fumarase activity have a growth defect on LB‐rich medium plates. To elucidate the reason for this phenotype, we cultivated the wild=type strain BP264 and the reconstituted mutants BP276 (*gltAB ansR citG*) and BP292 (*aspB ansR*) in LB and in brain heart infusion (BHI) liquid medium. As shown in Figure 7A,C, only the triple mutant BP276 lacking fumarase activity had a growth defect in LB medium. The fact that the addition of glucose to LB and BHI medium relieves the growth defect of the strain BP276 indicates that the block in the TCA cycle probably prevents the bacteria from efficiently using the amino acids that are present in LB and BHI‐rich media (Figure 7B,D). Next, we performed a short‐term evolution experiment by passaging the wild‐type strain BP264 and the *citG* mutants BP275 (*ansR citG*) and BP276 (*gltAB ansR citG*) for 10 days in LB liquid medium (see *Experimental procedures*). From each culture, we isolated a single colony on LB medium for further analysis. The derivatives of the strains BP264, BP275, and BP276 were designated as BP384, BP369 and BP370, respectively. As shown in Figure 7E, the wild‐type strain and its evolved derivative were phenotypically indistinguishable from each other. In contrast, the evolved *citG* mutants formed larger colonies than the parental strains (Figure 7E). The cultivation of the bacteria also showed that the lytic phenotype was less pronounced in the *citG* mutants (Figure 7F). Genome sequencing revealed that both evolved *citG* mutant strains had activated the cryptic *gudB* ^*CR*^ GDH gene (Belitsky & Sonenshein, 1998; Gunka et al., 2012; Zeigler et al., 2008). To conclude, the growth defect of the *citG* mutants can be partially suppressed by enhancing GDH activity, which is required for efficient utilization of amino acids of the glutamate family.

### Genomic adaptation of *citG* mutants to toxic levels of arginine

**FIGURE 8:**
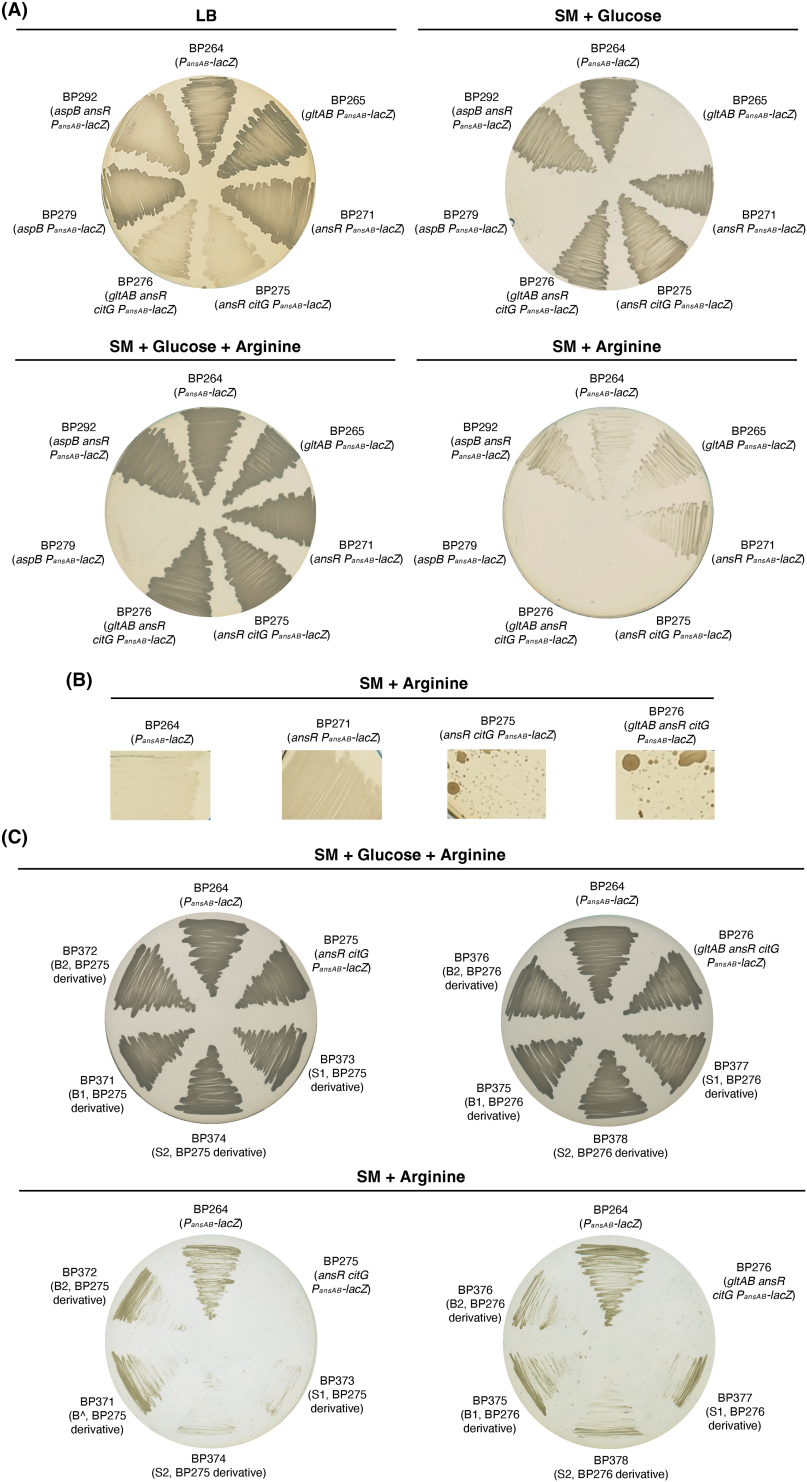
Adaptation of *Bacillus subtilis* fumarase mutants to arginine. (A) Growth of the strains BP264, BP265 (*gltAB*), BP271 (*ansR*), BP275 (*ansR citG*), BP276 (*gltAB ansR citG*), BP279 (*aspB*), and BP292 (*aspB ansR*) on LB and SM plates. Arginine and glucose were added to a final concentration of 0.5% (w/v). (B) Emergence of suppressor mutants derived from the fumarase mutants BP275 (*ansR citG*) and BP276 (*gltAB ansR citG*) on SM plates containing 0.5% (w/v) arginine. The plates were incubated for 10 days at 37°C. (C) Growth of the arginine adapted suppressor mutants BP371 (B1, BP275 derivative), BP372 (B2, BP275 derivative), BP373 (S1, BP275 derivative), and BP374 (S1, BP275 derivative), BP375 (B1, BP276 derivative), BP376 (B2, BP276 derivative), BP377 (S1, BP276 derivative), and BP378 (S2, BP276 derivative) on SM‐glucose plates without and with 0.5% (w/v) arginine. The strains BP264 (*P*_*ansAB*_‐*lacZ*), BP275 (*ansR citG*), and BP276 (*gltAB ansR citG*) served as controls. The plates were incubated for 48 h at 37°C. ‘S’ and ‘B’ indicate ‘small colony morphology’ and ‘big colony morphology’, respectively. Growth experiments using agar plates were carried out three times independently (*N* = 3).

**FIGURE 9:**
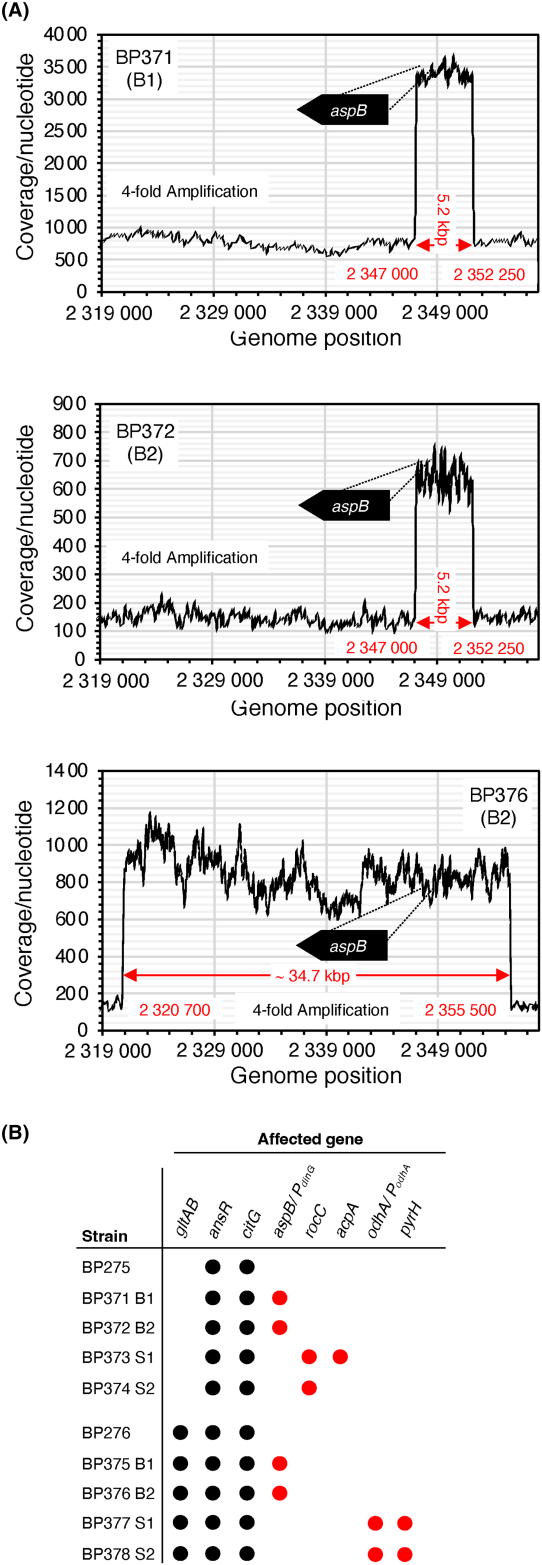
Genomic adaptation of *B. subtilis* fumarase mutants relieves arginine toxicity. (A) Read coverages along the chromosomal segment ranging from 2.319.000 to 2.358.000 bp. Based on the average coverage of the amplified regions and of the remaining genomes it can be inferred that four copies of the 5.2 and 37.4 kbp long regions containing the *aspB* gene are present in the suppressors BP371 (B1, BP275 derivative), BP372 (B2, BP275 derivative), and BP376 (B2, BP276 derivative). (B) Genes that are absent (black circles) or mutated (red circles) in the arginine adapted suppressor mutants BP371 (B1, BP275 derivative), BP372 (B2, BP275 derivative), BP373 (S1, BP275 derivative), and BP374 (S1, BP275 derivative), BP375 (B1, BP276 derivative), BP376 (B2, BP276 derivative), BP377 (S1, BP276 derivative), and BP378 (S2, BP276 derivative).

Previously, it has been shown that arginine is toxic for a *B. subtilis rocG gudB* mutants that cannot degrade glutamate (Belitsky & Sonenshein, 1998). We hypothesized that also an operating TCA cycle is needed to prevent the accumulation of glutamate and TCA cycle intermediates. Therefore, we assessed the growth of the *citG* mutants lacking fumarase activity. Arginine is taken up by *B. subtilis* via the permeases RocC and RocE and converted to glutamate (Belitsky & Sonenshein, 1998; Calogero et al., 1994; Gardan et al., 1995). For this purpose, we propagated the strains BP264, BP265 (*gltAB*), BP271 (*ansR*), BP275 (*ansR citG*), BP276 (*gltAB ansR citG*), BP279 (*aspB*), and BP292 (*aspB ansR*) on an LB plate (control) and on SM minimal medium plates supplemented with glucose, glucose plus arginine (0.5% (w/v)), or only arginine (0.5% (w/v)). The strains BP264, BP265, BP271, BP279, and BP292 served as controls. Both *citG* mutants (strains BP275 and BP276) showed the previously observed growth defect on the LB plate and no growth was visible on the SM plate containing only arginine or products of arginine degradation (Figure 8A). Thus, arginine is indeed toxic for the *citG* mutants, probably due to the accumulation of glutamate or TCA cycle intermediates. Moreover, arginine was not toxic for the bacteria in the presence of glucose because the cellular demand for glutamate is higher during growth with the preferred carbon source glucose (Figure 8A; Belitsky & Sonenshein, 1998; Commichau, Wacker, et al., 2007). When the arginine‐containing plates were further incubated for 48 h at 37°C, we observed that the *citG* mutants BP275 and BP276 formed small and large suppressor mutants, which was not the case for the other strains (Figure 8B; data not shown). Next, we selected two small and two large colonies that were derived from the two *citG* mutants and propagated the bacteria on SM plates supplemented with either glucose and arginine or only arginine (Figure 8C). The wild‐type strain BP264 and the parental strains BP275 (*ansR citG*) and BP276 (*gltAB ansR citG*) served as controls. As expected, all strains grew on the plates containing glucose that alleviates arginine toxicity. In contrast, the suppressor mutants BP371–BP374 and BP375–BP378 that were derived from the strains BP275 and BP276, respectively, showed slight but significant growth on the SM plates supplemented with arginine (Figure 8C). Genome sequencing revealed that the large suppressor mutants BP371, BP372, BP375, and BP376 had amplified genomic segments containing the *aspB* gene that likely results in enhanced expression of *aspB* (Table 2; Figure 9A). The strain BP375 has acquired a mutation in the *P*_*dinG*_ promoter that lies upstream of the *dinG, ypmA, ypmB*, and *aspB* genes. It is tempting to speculate that the mutation in the *P*_*dinG*_ promoter would enhance the expression of *dinG* including the *aspB* gene. Since AspB can convert glutamate and oxaloacetate to aspartate and 2‐oxoglutarate, the overproduction of the aspartate transaminase would prevent the accumulation of glutamate to toxic levels. Previously, it has indeed been observed that *a B. subtilis* mutant lacking GDH activity and AnsR can grow with glutamate as the sole source of carbon and nitrogen using AspB (Flórez et al., 2011). The two other suppressors BP373 and BP374 that were derived from the strain BP275 (*ansR citG P*_*ansAB*_ *‐lacZ*) and formed small colonies on arginine‐containing SM plates had accumulated mutations in the *rocC* gene encoding the arginine permease RocC (Figure 1A; Table 2). P411L exchange in RocC very likely reduces the activity of the arginine permease in strain BP373 because the residue P411 is located in a transmembrane helix (Figure S3A). Moreover, the suppressor BP374 carries a frameshift mutation in *rocC*. Thus, in addition to *aspB* amplification, the bacteria lacking CitG can adapt to toxic levels of glutamate by reducing arginine uptake. It remains elusive why BP373 carries a mutation in the *acpP* gene encoding the essential AcpA protein that is involved in fatty acid biosynthesis (Table 2; Figure S3B; Schujman et al., 2003). Moreover, we identified mutations in the *P*_*odhA*_ promoter region and in the *odhA* gene in the strains BP377 and BP378, respectively, that were derived from the strain BP276 (*gltAB ansR citG P*_*ansAB*_ *‐lacZ*; Table 2). The deletion in the *odhA* gene of the strain BP378 certainly inactivates the OdhAB‐PdhD 2‐oxoglutarate dehydrogenase. Therefore, the mutation in the *P*_*odhA*_ promoter region likely reduces the expression of *odhA*. Previously, it has been reported that the fitness of the *odhA* mutant is significantly enhanced during growth in minimal medium. Therefore, the inactivation or altered expression of *odhA* may not be required for adaptation to arginine (Koo et al., 2017). The strains BP377 and BP378 also carry mutations in the essential *pyrH* gene encoding the uridylate kinase PyrH that converts ATP and UMP to ADP and UDP (Table 2; Figure S3C; Quinn et al., 1991). To our surprise, we did not identify *gudB*‐activating mutations. It is tempting to speculate that AspB is more efficient in converting glutamate and oxaloacetate to aspartate and 2OG. To conclude, arginine toxicity can be relieved by *aspB* amplification and by mutations affecting arginine uptake. The relevance of the *odhA* and *pyrH* mutations for the adaptation to arginine needs to be tested in the future.

**TABLE 2:** Identified mutations in the evolved *ansR citG* and *ansR citG gltAB* mutants.

Strain	Mutant	Parental strain	Phenotype	Affected gene, mutations	Amino acid exchanges, effect on the protein
Mutations in the strains BP275 (*ansR citG P*_*ansAB*_ *‐lacZ*) and BP276 (*gltAB ansR citG P*_*ansAB*_ *‐lacZ*) evolved in LB medium					
BP384	LBW^a^	BP264 (*P*_*ansAB*_ *‐lacZ*)	Growth in LB	*–*	–
BP369	LB1^a^	BP275 (*ansR citG P*_*ansAB*_ *‐lacZ*)	Growth in LB	*gudB* ΔG279‐C287	Synthesis of GudB1
BP370	LB2^a^	BP276 (*gltAB ansR citG P*_*ansAB*_ *‐lacZ*)	Growth in LB	*gudB* ΔG279‐C287	Synthesis of GudB1
Mutations reducing arginine toxicity of the strains BP275 (*ansR citG P*_*ansAB*_ *‐lacZ*) and BP276 (*gltAB ansR citG P*_*ansAB*_ *‐lacZ*)					
BP371	B1^a^	BP275 (*ansR citG P*_*ansAB*_ *‐lacZ*)	Growth on SM‐Arg, big colonies	5.2 kbp amplification including *aspB*	Increased AnsB synthesis
BP372	B2^a^	BP275 (*ansR citG P*_*ansAB*_ *‐lacZ*)	Growth on SM‐Arg, big colonies	5.2 kbp amplification including *aspB*	Increased AnsB synthesis
BP373	S1^a^	BP275 (*ansR citG P*_*ansAB*_ *‐lacZ*)	Growth on SM‐Arg, small colonies	*acpA* G55T *rocC* C1232T	AcpA D19Y, RocC P411L
BP374	S2^a^	BP275 (*ansR citG P*_*ansAB*_ *‐lacZ*)	Growth on SM‐Arg, small colonies	*rocC* + T698	RocC ΔC270
BP375	B1^a^	BP276 (*gltAB ansR citG P*_*ansAB*_ *‐lacZ*)	Growth on SM‐Arg, big colonies	*P*_*dinG*_ (C‐63 T)	Increased AspB synthesis
BP376	B2^a^	BP276 (*gltAB ansR citG P*_*ansAB*_ *‐lacZ*)	Growth on SM‐Arg, big colonies	34.7 kbp amplification including *aspB*	Increased AspB synthesis
BP377	S1^a^	BP276 (*gltAB ansR citG P*_*ansAB*_ *‐lacZ*)	Growth on SM‐Arg, small colonies	*odhA* ΔT175‐A351, *pyrH* A433C	OdhA not synthesized, PyrH T145P
BP378	S2^a^	BP276 (*gltAB ansR citG P*_*ansAB*_ *‐lacZ*)	Growth on SM‐Arg, small colonies	*P*_*odhA*_ (G‐189A), *pyrH* A433C	Altered *odhA* expression? PyrH T145P

^a^ Mutations were identified by genome sequencing.

## DISCUSSION

Here, we found that the glutamate auxotrophy of a *B. subtilis gltAB* mutant lacking GOGAT activity can be relieved by the mutational inactivation of the *ansR* and *citG* genes. The de‐repression and the inactivation of the *ansB* and *citG* genes, respectively, allow the *gltAB* mutant to synthesize glutamate via the L‐aspartase/L‐aspartate transaminase‐dependent route (Figure 1A,D). Moreover, the inactivation of the *ansR* gene restores the aspartate prototrophy of an *aspB* mutant. It should be noted that in both strains and the derived suppressor mutants, GS is still used to assimilate ammonium in addition to AnsB.

In addition to the inactivation of the *gltAB* genes, also other genetic lesions can lead to glutamate auxotrophy in *B. subtilis*. For instance, the inactivation of the *gltC* gene encoding the DNA‐binding regulator GltC that is required for the transcriptional activation of the *gltAB* genes results in glutamate auxotrophy (Figure 1B; Bohannon & Sonenshein, 1989; Dormeyer et al., 2017). As observed in this study, glutamate auxotrophy can be quickly relieved. The expression of the *gltAB* genes can be restored by (i) a gain‐of‐function mutation in the *gltR* gene, encoding a LysR‐type transcription factor of unknown function (Belitsky & Sonenshein, 1997; Dormeyer et al., 2017), (ii) by a promoter‐up mutation in the *P*_*gltAB*_ promoter, and (iii) by the selective amplification of a chromosomal segment containing the *gltAB* genes (Dormeyer et al., 2017). It has also been shown that a *B. subtilis ccpA* mutant is auxotrophic for glutamate due to insufficient expression of the *gltAB* genes and de‐repression of the *rocG* GDH gene that is repressed by CcpA during growth with glucose (Belitsky et al., 2004; Commichau, Wacker, et al., 2007; Faires et al., 1999; Wacker et al., 2003). Strikingly, even in the absence of the pleiotropic transcription factor CcpA, *B. subtilis* may restore glutamate biosynthesis by the accumulation of mutations in the *topA* gene encoding the essential DNA topoisomerase I TopA (Reuß et al., 2018). The TopA mutant variants cause glutamate prototrophy of the *ccpA* mutant due to enhanced relaxation of the chromosomal DNA, which results in the re‐organization of the global transcription network, re‐routing of central metabolism and in the inactivation of the GDH (Reuß et al., 2018). Thus, the glutamate auxotrophy in *B. subtilis* in a minimal medium (i.e., in the absence of other amino acids) can be relieved by mutations restoring the expression of the *gltAB* genes or by the mutational activation of an alternative de novo glutamate biosynthesis route.

The present work raises the question of whether the GS‐GOGAT‐dependent biosynthesis pathway is the dominant pathway for the biosynthesis of the major cellular amino group donor in nature because the non‐canonical fumarate‐based ammonium assimilation pathway does not depend on ATP and NADPH_2_ (Figure 1D). Like in *B. subtilis*, also in *E. coli*, the overexpression of the native L‐aspartase can replace the canonical glutamate‐based ammonium assimilation pathway (Schulz‐Mirbach et al., 2022). To address the question of how widespread the coding capacity for the bypass is, we performed a bioinformatic survey. We used the GltAB, AnsB, AspB, and CitG proteins from *B. subtilis* as queries to screen all annotated proteins from 14,954 bacterial genomes. This screen revealed that >1600 genomes lack homologues for GltA, GltB, and a GudB/RocG but possess AnsB, AspB, and CitG homologues (Figure S4). It will be interesting to test whether the L‐aspartase/L‐aspartate transaminase‐dependent route is the major glutamate biosynthesis pathway in some of these species. Previously, it has been reported that a GOGAT/GDH‐deficient *Corynebacterium glutamicum* strain still grows with glucose and ammonium as single sources of carbon and nitrogen, respectively (Figure S5; Rehm et al., 2010). However, the Aspartase AspA, which shares 46.7% overall sequence identity with AnsB from *B. subtilis*, is not involved in the fumarate‐based ammonium assimilation pathway because a *C. glutamicum* mutant lacking GOGAT and GDH activity as well as AspA still grows on minimal medium plates containing only glucose and ammonium (Figure S5). This suggests the existence of another, yet unknown route for ammonium assimilation in this organism.

In *B. subtilis*, the GDHs are not involved in ammonium assimilation (Belitsky & Sonenshein, 1998; Commichau et al., 2008). Similarly, in plants, the GDHs do not significantly participate in glutamate formation (Lea & Miflin, 2003). In plants, two types of GOGATs are involved in de novo synthesis of glutamate: NADPH_2_‐ and ferredoxin‐(Fd)‐dependent GOGATs. The GOGATs play distinct roles in different plant tissues and during various phases of growth and development (Suzuki & Knaff, 2005). It has been observed that in *Arabidopsis thaliana*, overexpressing Fd‐dependent GOGAT under non‐photorespiratory conditions, glutamate, and several other amino acids increased (Ishizaki et al., 2010). It will be interesting to test whether ammonium assimilation in *B. subtilis* can be enhanced in a strain in which the canonical glutamate‐based and non‐canonical fumarate‐based ammonium assimilation pathways are active. However, it is tempting to speculate that multiple genomic changes are necessary to prevent the cell from maintaining carbon and nitrogen metabolism in balance.

As described above, glutamate biosynthesis and degradation are tightly regulated depending on the carbon and nitrogen sources that are available for *B. subtilis* (Figure 1; Gunka & Commichau, 2012). Here, we observed that the *B. subtilis* strain relying on the L‐aspartase/L‐aspartate transaminase‐dependent route for glutamate biosynthesis has a growth defect in rich medium and in the presence of arginine. The growth defect can be relieved by increased synthesis of AspB, by reduced flux through the TCA cycle and arginine uptake. In the future, it will be interesting to investigate whether the *B. subtilis* strain using the alternative glutamate biosynthesis route can be evolved in such a way that it robustly grows during nitrogen limitation and in the presence of arginine.

## EXPERIMENTAL PROCEDURES

### Bacterial strains, chemicals, and DNA manipulation

Bacterial strains used in this study are listed in Table S1. Primers were purchased from Sigma‐Aldrich (Munich, Germany) and are listed in Table S2. Chemicals and media were purchased from Sigma‐Aldrich (Munich, Germany), Carl Roth (Karlsruhe, Germany) and Becton Dickinson (Heidelberg, Germany). Bacterial chromosomal DNA was isolated using the peqGOLD bacterial DNA kit (Peqlab, Erlangen, Germany). PCR products were purified using the PCR purification kit (Qiagen, Germany). Phusion DNA polymerase was purchased from Thermo Scientific (Germany) and used according to the manufacturer's instructions.

### Cultivation of bacteria

Bacteria were grown in lysogeny broth (LB) (Sezonov et al., 2007) and brain heart infusion (BHI) (Rosenow, 1919)‐rich medium or in Spizizen (SM) (Anagnostopoulos & Spizizen, 1961), C‐Glc (Commichau, Herzberg, et al., 2007; Commichau, Wacker, et al., 2007; Dormeyer et al., 2019), and CGXII (Keilhauer et al., 1993) minimal medium. Agar plates were prepared with 15 g agar/l (Roth, Germany). Growth in liquid medium was monitored using 96‐well plates (Microtest Plate 96‐Well, F Sarstedt, Germany) at 37°C and medium orbital shaking at 237 cpm (4 mm) in a Synergy H1 plate reader (Agilent, USA) equipped with the Gen5 software, and the OD_600_ was measured in 10–15 min intervals. Single colonies were used to inoculate 5 mL overnight LB cultures that were incubated at 220 rpm and 30°C. The OD_600_ was adjusted to 0.1, and 150 μL of the cell suspensions were transferred into 96‐well plates. Bacteria were cultivated in the Synergy H1 plate reader as described above.

### Plasmid and strain construction

The plasmids used and generated in this study are listed in Table S3. The plasmids pBP1110 and pBP1111 carrying the carrying the *P*_*ansAB*_ promoter and the *ansR* gene together with the *P*_*ansAB*_ promoter, respectively, were constructed as follows. The *P*_*ansAB*_ promoter and the *ansR‐P*_*ansAB*_ promoter fragments were amplified by PCR using the primer pairs SM1/SM2 and SM36/SM2, respectively, digested with *Eco*RI and *Bam*HI and ligated to pAC7 (Weinrauch et al., 1991) that was cut with the same enzymes. The generated plasmids were verified by Sanger sequencing (Microsynth‐SeqLab Sequence Laboratories).

Deletion of the *ansAB, ansR, aspB, citG, gltAB*, and *recN* genes in *B. subtilis* was achieved by transformation with long‐flanking homology (LFH) PCR products constructed using oligonucleotides (Table S2) to amplify DNA fragments flanking the target gene and the intervening *spc* spectinomycin, *cat* chloramphenicol, *ermC* erythromycin/lincomycin, and *tet* tetracycline resistance genes from the plasmids pDG1726, pGEM‐cat, pDG647, and pDG1514 (Guérout‐Fleury et al., 1995), respectively, as described previously (Gaballa et al., 2010). When required, antibiotics 5‐bromo‐4‐chloro‐3‐indolyl‐β‐D‐galactopyranoside (X‐Gal) were added to the following concentrations: ampicillin (100 μg/mL), kanamycin (10 μg/mL), chloramphenicol (5 μg/mL), spectinomycin (150 μg/mL), erythromycin and lincomycin (2 and 25 μg/mL), and X‐Gal (100 μg/mL). *B. subtilis* was transformed with plasmids, PCR products and with chromosomal DNA according to a previously described two‐step protocol (Kunst & Rapoport, 1995). AmyE amylase activity was detected after growth on agar plates containing nutrient broth (7.5 g/L) Bacto agar (17 g/L; Difco) and hydrolysed starch (5 g/L; Connaught). Starch degradation was detected by sublimating iodine onto the plates.

### Genome sequencing

Genomic DNA was prepared from 500 μL overnight cultures using the MasterPure Complete DNA & RNA Purification Kit (Lucigen, Middleton, USA) following the instruction of the manufacturer with the modification of physically opening cells with the TissueLyser II (Qiagen). Purified genomic DNA was paired end sequenced (2 × 150 bp) (GENEWIZ). The reads were mapped onto the *B. subtilis* reference genome NC_000964 from GenBank (Barbe et al., 2009) as previously described (Widderich et al., 2016) using the Geneious software package (Biomatters Ltd.; Kearse et al., 2012). All identified mutations were verified by performing PCRs and Sanger sequencing.

### Metabolomics

The *B. subtilis* strains were grown overnight in 4 mL LB medium at 28°C and 160 rpm. The overnight cultures were used to inoculate 100 mL shake flasks containing 10 mL MSSM medium to an OD_600_ of 0.1. The cultures were incubated at 37°C and 160 rpm. 0.5 mg biomass were collected using a PVDF filter (0.45 μm pore site) in a glass frit. The cells on the filters were resuspended in 1 mL ice‐cold extraction solution (acetonitrile/methanol/ultrapure water, 40%/40%/20%) and incubated for 1 h at −20°C. The cell extracts were centrifuged for 15 min and 20,000 *g* at −9°C, and stored at –80°C until further processing. Relative concentrations of the metabolites Glu, Gln, Asp, Asn, Cit, Suc, and Mal in the cell extracts were measured via isotope ratio LC–MS/MS as described previously (Guder et al., 2017).

### Evolution of *B. subtilis* in LB medium

A total of 100 mL shake flasks containing 10 mL LB medium were inoculated with a single colonies of the *B. subtilis* strains BP264, BP275, and BP276 and cultivated for 24 h at 37°C. Next day, 100 μL of the cultures were used to inoculate a fresh shake flask. This step was repeated 10 times. Single colonies were isolated by propagating the aliquots of the cultures on LB plates. After phenotypic inspection, one derivative of each strain (Figure 7E) was subjected to genome sequencing.

### Identification of bacterial genomes lacking the *B. subtilis* GOGAT

Annotated proteins from all available bacterial genomes were downloaded from RefSeq (14,954 genomes as of 13.04.2022). We used BLASTP searches to identify genomes lacking GltAB and GudB but possessing AnsB, AspB, and CitG. In practice, bacterial genomes with the *B. subtilis* GltA and GltB subunits were identified, and the enzyme was considered to be absent if none of the subunits had a significant BLASTP hit. *B. subtilis* proteins were used as queries and an e‐value threshold of e‐50 was applied, experimentally tested on several bacterial species. A total of 2791 genomes were found to lack GltAB, which were subjected to further BLASTP searches to identify AnsB, AspB, and CitG using the *B. subtilis* protein queries (*e*‐value < e‐50). Some 1642 genomes lacked GltAB and GudB but had AnsB, AspB, and CitG homologues. The taxonomic lineage of these genomes was obtained from NCBI through the use of taxIDs in ETE3 (Huerta‐Cepas et al., 2016).

## AUTHOR CONTRIBUTIONS

**Mohammad Saba Yousef Mardoukhi:** Formal analysis (equal); investigation (equal); methodology (equal); validation (equal); visualization (equal). **Johanna Rapp:** Formal analysis (equal); methodology (equal). **Iker Irisarri:** Formal analysis (equal); investigation (equal); software (equal); writing – original draft (equal). **Katrin Gunka:** Investigation (equal); methodology (equal). **Hannes Link:** Funding acquisition (equal); methodology (equal). **Jan Marienhagen:** Investigation (equal); methodology (equal). **Jan de Vries:** Investigation (equal); software (equal); supervision (equal). **Jörg Stülke:** Funding acquisition (equal); supervision (equal). **Fabian M. Commichau**: Funding acquisition (equal); visualization (equal); formal analysis (equal); writing (equal); supervision (equal).

## CONFLICT OF INTEREST STATEMENT

The authors declare that there is no potential conflict of interest.

## Supporting information


Figure S1.
Figure S2.
Figure S3.
Figure S4.
Figure S5.
Table S1.
Table S2.
Table S3.
