# Supplementary material for: Metabolic rewiring enables ammonium assimilation via a non‐canonical fumarate‐based pathway
Source: Microb Biotechnol. 2024 Mar 14;17(3):e14429. doi: 10.1111/1751-7915.14429 (PMC10938345; doi:10.1111/1751-7915.14429)
Supplement: Supplementary file 1 — Figure S1. Figure S2. Figure S3. Figure S4. Figure S5. Table S1. Table S2. Table S3. [file MBT2-17-e14429-s001.docx]

**Metabolic rewiring enables ammonium assimilation via a non-canonical fumarate-based pathway**

**Mohammad Saba Yousef Mardoukhi^1^, Johanna Rapp^2^, Iker Irisarri^3,4^, Katrin Gunka^5^, Hannes Link^2^, Jan Marienhagen^6,7^, Jan de Vries^3,4^, Jörg Stülke^5^, Fabian M. Commichau^1*^**

^1^FG Molecular Microbiology, Institute for Biology, University of Hohenheim, 70599 Stuttgart, Germany

^2^Interfaculty Institute for Microbiology and Infection Medicine Tübingen, University of Tübingen, Germany

^3^Department of Applied Bioinformatics, Institute of Microbiology & Genetics, GZMB, Georg-August-University Göttingen, 37077 Göttingen, Germany

^4^Campus Institute Data Science, University of Göttingen, 37077 Göttingen, Germany

^5^Department of General Microbiology, Institute for Microbiology & Genetics, GZMB, Georg-August-University Göttingen, 37077 Göttingen, Germany

^6^Institute of Bio- and Geosciences, IBG-1: Biotechnology, Forschungszentrum Jülich, D-52425 Jülich, Germany

^7^Institut of Biotechnology, RWTH Aachen University, Worringer Weg 3, D-52074 Aachen, Germany

For correspondence. fabian.commichau@uni-hohenheim.de

University of Hohenheim

Garbenstrasse 30

70599 Stuttgart

Germany

Phone: +49-711-459-22222; Fax: +49-711-459-22238

Fabian.commichau@uni-hohenheim.de

**Content**

Figure S1

Figure S2

Figure S3

Figure S4

Figure S5

Table S1. Strains

Table S2. Primers

Table S3. Plasmids

**Supporting information references**

**Fig. S1. A.** Growth of the *gltAB* suppressor mutants BP364 (M1) and BP365 (M2), and the parental strain BP261 (*gltAB*) on CGXII plates without and with 0.5% (w/v) glutamate. The plates were incubated for 48 h at 37°C. **B.** Verification of the replacement of the *gltAB* genes by the *tet* resistance gene in the suppressors BP364 (M1) and BP365 (M2). Chromosomal DNA of the *gltAB* mutant GP807 served as the control. **C.** Localization of amino acid exchanges in a structure model of AnsR. The model was generated using the Swiss-model server for homology modeling of protein structures [Waterhouse et al., 2018] and a model of the EspR transcription factor from *Mycobacterium tuberculosis* (PDBid: 3QF3) [Blasco et al., 2011]. **D.** Mutation in the ribosome binding site (RBS) of the *P_ansR_* promoter in the suppressor mutant BP287 that was derived from the strain BP265 (*gltAB P_ansAB_-lacZ*). **E** and **F**, localization of amino acid exchanges in a structure model of CitG and AnsA, respectively. The models were generated as described for the AnsR model using structures of the *E. coli* fumarase (PDBid: 6P3C) and the *Thermococcus kodakarensis* L-asparaginase (PDBid: 5Ot0) [Guo et al., 2017].

**Fig. S2.** Emergence of suppressor mutants of the strain BP265 (*gltAB P_ansAB_-lacZ*) after 8 days of incubation at 37°C on C-Glc medium plates containing the indicated supplements. All plates contained X-Gal.

**Fig. S3. A.** Localization of the P411L exchange in RocC of the suppressor mutant BP373. The RocC topology model was created with Protter [Omasists et al., 2014]. **B** and **C**, localization of amino acid exchanges in a structure model of the AcpA-AcpS complex and of PyrH, respectively. The models were generated as described for the AnsR model using structures of *B. subtilis* AcpA-AcpS (PDBid: 1F80) [Parris et al., 2000] and the *E. coli* UMP kinase (PDBid: 2V4Y) [Meyer et al., 2008].

**Fig. S4.** Taxonomic distribution of bacterial species lacking GltA, GltB and GudB/RocG homologs and possessing AnsB, AspB and CitG homologs, thus suggesting a possible metabolic bypass (in a total of 1642 from 14954 investigated genomes).

**Fig. S5.** Growth of the *C. glutamicum* wild type and the *gltB gdh*, *gltB gdh aspT* and *gltB gdh aspA* mutants on CGXII medium in the absence and in the presence of glutamate (0.5% (w/v)) or casamino acid hydrolysate (CAA, 0.1% (w/v)). The plates were incubated for 48 h at 30°C. The *C. glutamicum* aminotransferases AspT and AspA share 24% and 46.7% overall amino acid sequence identity, respectively, with the *B. subtilis* AspB and AnsB proteins respectively.

**Table S1.** Strains

| **Strain** | **Bacterium, mutant** | **Genotype** | **Reference, construction^a^** |
| --- | --- | --- | --- |
| SP1 | *B. subtilis* | Prototrophic derivative of strain 168 | Richts et al., 2020 |
| 168 | *B. subtilis* | *trpC2* | Laboratory strain collection |
| GP807 | *B. subtilis* | *trpC2 gltAB::tet* | LFH → 168 |
| GP1153 | *B. subtilis* | *trpC2 ansAB::ermC* | LFH → 168 |
| BP234 | *B. subtilis* | *trpC gltP::cat* | Wicke et al., 2019 |
| BP261 | *B. subtilis* | *gltAB::tet* | cDNA GP807 → SP1 |
| BP264 | *B. subtilis* | *amyE::*(*P_ansAB_-lacZ aphA*) | pBP1110 → SP1 |
| BP265 | *B. subtilis* | *amyE::*(*P_ansAB_-lacZ aphA*)  *gltAB::tet* | pBP1110 → BP261 |
| BP266 | *B. subtilis* | *ansR::cat* | LFH → SP1 |
| BP267 | *B. subtilis* | *citG::ermC* | LFH → SP1 |
| BP269 | *B. subtilis* | *ansAB::ermC* | cDNA GP1153 → SP1 |
| BP270 | *B. subtilis* | *aspB::spc* | LFH → SP1 |
| BP271 | *B. subtilis* | *amyE::*(*P_ansAB_-lacZ aphA*) *ansR::cat* | cDNA BP266 → BP264 |
| BP272 | *B. subtilis* | *amyE::*(*P_ansAB_-lacZ aphA*) *citG::ermC* | cDNA BP267 → BP264 |
| BP273 | *B. subtilis* | *amyE::*(*P_ansAB_-lacZ aphA*) *ansR::cat gltAB::tet* | cDNA BP266 → BP265 |
| BP274 | *B. subtilis* | *amyE::*(*P_ansAB_-lacZ aphA*) *citG::ermC gltAB::tet* | cDNA BP267 → BP265 |
| BP275 | *B. subtilis* SP1 | *amyE::*(*P_ansAB_-lacZ aphA*) *ansR::cat citG::ermC* | cDNA BP271 → BP272 |
| BP276 | *B. subtilis* SP1 | *amyE::*(*P_ansAB_-lacZ aphA*) *ansR::cat citG::ermC* | cDNA BP273 → BP274 |
| BP279 | *B. subtilis* SP1 | *amyE::*(*P_ansAB_-lacZ aphA*) *aspB::spc* | cDNA BP270 → BP264 |
| BP280 | *B. subtilis* SP1 | *gltAB::tet ansAB::ermC* | cDNA BP269 → BP261 |
| BP281 | Suppressor of BP265 | *amyE::*(*P_ansAB_-lacZ aphA*) *gltAB::tet ansR* +A36 *citG* +G917 | Selection |
| BP282 | Suppressor of BP265 | *amyE::*(*P_ansAB_-lacZ aphA*) *gltAB::tet ansR* Δ2456934-2457298 | Selection |
| BP283 | Suppressor of BP265 | *amyE::*(*P_ansAB_-lacZ aphA*) *gltAB::tet ansR* +A36 *citG* C1226A | Selection |
| BP284 | Suppressor of BP265 | *amyE::*(*P_ansAB_-lacZ aphA*) *gltAB::tet ansR* +A36 | Selection |
| BP285 | Suppressor of BP265 | *amyE::*(*P_ansAB_-lacZ aphA*) *gltAB::tet ansR* +A36 | Selection |
| BP286 | Suppressor of BP265 | *amyE::*(*P_ansAB_-lacZ aphA*) *gltAB::tet ansR* +A36 | Selection |
| BP287 | Suppressor of BP265 | *amyE::*(*P_ansAB_-lacZ aphA*) *gltAB::tet P_ansAB_* (G-10A) *citG* T876A | Selection |
| BP288 | Suppressor of BP265 | *amyE::*(*P_ansAB_-lacZ aphA*) *gltAB::tet ansR* +A36 | Selection |
| BP289 | Suppressor of BP265 | *amyE::*(*P_ansAB_-lacZ aphA*) *gltAB::tet ansR* +A36 *citG* Δ3390211-3390276 | Selection |
| BP290 | Suppressor of BP265 | *amyE::*(*P_ansAB_-lacZ aphA*) *gltAB::tet ansR* +A36 | Selection |
| BP292 | *B. subtilis* | *amyE::*(*P_ansAB_-lacZ aphA*) *ansR::cat aspB::spc* | cDNA BP270 → BP271 |
| BP294 | *B. subtilis* | *amyE::*(*ansR-P_ansR_-lacZ aphA*)  *gltAB::tet* | pBP1111 → BP261 |
| BP296 | Suppressor of BP279 | *amyE::*(*P_ansAB_-lacZ aphA*) *aspB::spc citG* C686T *ansA* T43A | Selection |
| BP297 | Suppressor of BP279 | *amyE::*(*P_ansAB_-lacZ aphA*) *aspB::spc citG* C312G *ansA* T43A | Selection |
| BP298 | Suppressor of BP265 | *amyE::*(*P_ansAB_-lacZ aphA*) *gltAB::tet citG* A1033T 25.2 kbp amplification including *ansAB* | Selection |
| BP364 | Suppressor of BP261 | *gltAB::tet ansR* T302C 10.7 kbp deletion including *citG* | Selection |
| BP365 | Suppressor of BP261 | *gltAB::tet ansR* T169C *citG* ΔG62 | Selection |
| BP366 | Suppressor of BP279 | *amyE::*(*P_ansAB_-lacZ aphA*) *aspB::spc ansR* C171A | Selection |
| BP367 | Suppressor of BP279 | *amyE::*(*P_ansAB_-lacZ aphA*) *aspB::spc ansR* G3A | Selection |
| BP368 | Suppressor of BP279 | *amyE::*(*P_ansAB_-lacZ aphA*) *aspB::spc ansR* G94C | Selection |
| BP369 | Derivative of BP275 | *amyE::*(*P_ansAB_-lacZ aphA*) *ansR::cat citG::ermC gudB* ΔG279-C287 | Selection |
| BP370 | Derivatives of BP276 | *amyE::*(*P_ansAB_-lacZ aphA*) *gltAB::tet ansR::cat citG::ermC gudB* ΔG279-C287 | Selection |
| BP371 | Suppressor of BP275 | *amyE::*(*P_ansAB_-lacZ aphA*) *ansR::cat citG::ermC* 5.2 kbp amplification including *aspB* | Selection |
| BP372 | Suppressor of BP275 | *amyE::*(*P_ansAB_-lacZ aphA*) *ansR::cat citG::ermC* 5.2 kbp amplification including *aspB* | Selection |
| BP373 | Suppressor of BP275 | *amyE::*(*P_ansAB_-lacZ aphA*) *ansR::cat citG::ermC rocC* C1232T | Selection |
| BP374 | Suppressor of BP275 | *amyE::*(*P_ansAB_-lacZ aphA*) *ansR::cat citG::ermC rocC* + T698 | Selection |
| BP375 | Suppressor of BP276 | *amyE::*(*P_ansAB_-lacZ aphA*) *gltAB::tet ansR::cat citG::ermC P_dinG_* G-63A | Selection |
| BP376 | Suppressor of BP276 | *amyE::*(*P_ansAB_-lacZ aphA*) *gltAB::tet ansR::cat citG::ermC* 34.7 kbp amplification including *aspB* | Selection |
| BP377 | Suppressor of BP276 | *amyE::*(*P_ansAB_-lacZ aphA*) *gltAB::tet ansR::cat citG::ermC odhA* ΔT175-A351 | Selection |
| BP378 | Suppressor of BP276 | *amyE::*(*P_ansAB_-lacZ aphA*) *gltAB::tet ansR::cat citG::ermC P_odhA_* G-189A | Selection |
| BP382 | *B. subtilis* | *amyE::*(*ansR-P_ansR_-lacZ aphA*) | pBP1111 → SP1 |
| BP383 | *B. subtilis* | *amyE::*(*ansR-P_ansR_-lacZ aphA*)  *aspB::spc* | cDNA BP270 → BP382 |
| BP384 | Derivative of BP264 | *amyE::*(*P_ansAB_-lacZ aphA*) | Selection |
| BP647 | *B. subtilis* | *trpC recN::ermC* | LFH → 168 |
| BP1303 | *B. subtilis* | *trpC gdpP::spc* | Schwedt et al., 2023 |
| XL1-Blue | *E. coli* XL1-Blue | *recA1, endA1, gyrA96, thi-1, hsdR17, supE44, relA1, lac* [F ́ *proAB, lacI*q*Z*Δ*M15,* Tn*10* (Tet^r^)] | Stratagene |
| Wild type | *C. glutamicum* ATCC13032 | *-* | Abe et al., 1967 |
| Δ*gdh* Δ*gltB* | *C. glutamicum* ATCC13032 | Δ*gdh* Δ*gltB* | This study |
| Δ*gdh* Δ*gltB* Δ*aspA* | *C. glutamicum* ATCC13032 | Δ*gdh* Δ*gltB* Δ*aspA* | This study |
| Δ*gdh* Δ*gltB* Δ*aspT* | *C. glutamicum* ATCC13032 | Δ*gdh* Δ*gltB* Δ*aspT* | This study |

^a^ Arrows indicate strain construction by transformation.

**Table S2.** Primers

| **Primer** | **Description^a^** | **Purpose** |
| --- | --- | --- |
| FC75 | 5‘-CGAGCGCCTACGAGGAATTTGTATCGGAAGTGGC GCGTGAAGTGGATC | Construction of the strain GP807 |
| FC76 | 5‘-GGTTCTGACGGCGCGGGTATC | Construction of the strain GP807 |
| FC350 | 5‘-CAGCGAACCATTTGAGGTGATAGGCGGCAATAGT  TACCCTTATTATCAAG | Amplification of the *cat* gene |
| FC352 | 5‘-CGATACAAATTCCTCGTAGGCGCTCGGTTATAAAA  GCCAGTCATTAGGCCTATC | Amplification of the *cat* gene |
| FC356 | 5‘-CAGCGAACCATTTGAGGTGATAGGGATCCTTTAA  CTCTGGCAACCCTC | Amplification of the *ermC* gene |
| FC357 | 5‘-CGATACAAATTCCTCGTAGGCGCTCGGGCCGACT  GCGCAAAAGACATAATCG | Amplification of the *ermC* gene |
| FC359 | 5‘-CAGCGAACCATTTGAGGTGATAGGGACTGGCTCG  CTAATAACGTAACGTGACTGGCAAGAG | Amplification of the *spc* gene |
| FC361 | 5‘-CGATACAAATTCCTCGTAGGCGCTCGGTTTCCACC  ATTTTTTCAATTTTTTTATAATTTTTTT | Amplification of the *spc* gene |
| FC363 | 5‘-CGATACAAATTCCTCGTAGGCGCTCGGGAACTCTC  TCCCAAAGTTGATCCC | Verification of the integration of the *tet* gene |
| KG1 | 5‘-CCTATCACCTcaaatggttcgggccgattccgca tgcatcatgttc | Construction of the strain GP807 |
| KG2 | 5‘-cattcgcggaaggcgcaagctc | Construction of the strain GP807 |
| KG28 | 5‘-ATGGCTTGGACCCGTTATTGGGG | Construction of the strain GP1153 |
| KG29 | 5‘-CCTATCACCTCAAATGGTTCGCTGGAGCCAGCCCA TTTTCCCCTTC | Construction of the strain GP1153 |
| KG30 | 5‘-CCGAGCGCCTACGAGGAATTTGTATCGCGGCGCT GATCATCTTGTTGATG | Construction of the strain GP1153 |
| KG31 | 5‘-AAGTCGGCACAACGCCTCCGG | Construction of the strain GP1153 |
| MD56 | 5‘-AAAGTCGACTTATTGATACTGCTCCAGCTTAGAGA  AAAATTGAATG | Verification of the integration of the *tet* gene |
| MD119 | 5‘-CCTATCACCTCAAATGGTTCGCTGGACTTAACGAA ACGCCATGC | Construction of strain BP267 |
| MD120 | 5‘-ACCCGATTCTGTATTTGCCTTCT | Construction of strain BP267 |
| MD121 | 5‘-CCGAGCGCCTACGAGGAATTTGTATCGCCGCGTT CAAAAGAAACCGT | Construction of strain BP267 |
| MD122 | 5‘-AATCACGGGAGGAGACGGA | Construction of strain BP267 |
| mls fwd (kan) | 5‘-CAGCGAACCATTTGAGGTGATAGGGATCCTTTAAC TCTGGCAACCCTC | Amplification of the *ermC* gene |
| mls rev (kan) | 5‘-CGATACAAATTCCTCGTAGGCGCTCGGG CCGACTGCGCAAAAGACATAATCG | Amplification of the *ermC* gene |
| SM1 | 5‘-TTTGAATTCGAACTTCCGCTCCTTTTTCACC | Construction of pBP1110 |
| SM2 | 5‘-TTTGGATCCATACCATGCACCTCTTCACTGTATC | Construction of pBP1110, pBP1111 |
| SM3 | 5‘-TTTATGGAATACAGAATTGAACGAGAC | Amplification of *citG* |
| SM4 | 5‘-TTTTACGCCTTTGGTTTTACCATG | Amplification of *citG* |
| SM5 | 5‘-TTTCTAGCGCCCACATCAATTTTGGC | Construction of strain BP266 |
| SM6 | 5‘-TTTTCTTCGCCTTCTTCAAGACATTG | Construction of strain BP266 |
| SM7 | 5‘-CCTATCACCTCAAATGGTTCGCTGGAACTTCCGCT  CCTTTTTCACCTTGAG | Construction of strain BP266 |
| SM8 | 5‘-CCGAGCGCCTACGAGGAATTTGTATCGATCTTTAG  CTCACGGTTTAATTTTA | Construction of strain BP266 |
| SM9 | 5‘-TTTTCATCTGGAAAATATCGCGAGCTTGACG | Construction of strain BP266 |
| SM10 | 5‘-TTTCGCCAAAACATTAACGCTGGACAGAAT | Construction of strain BP266 |
| SM11 | 5‘-TTTCCACACGGCCGTTAAACAGGG | Construction of strain BP267 |
| SM12 | 5‘-TTTGCTGGATATTTTTAAGCCGCGCC | Construction of strain BP267 |
| SM13 | 5‘-CCTATCACCTCAAATGGTTCGCTGTTATGTATCCC  TCCATAACGGTTGCTTC | Construction of strain BP267 |
| SM14 | 5‘-CCGAGCGCCTACGAGGAATTTGTATCGATAGGAA  GAACGGCTGCTTTTTAAG | Construction of strain BP267 |
| SM15 | 5‘-TTTTGGTCATATCCTAGCAGGCCTCCG | Construction of strain BP267 |
| SM16 | 5‘-TTTCGTCCAATTCTCTCATTCTAGATTCACCCT | Construction of strain BP267 |
| SM18 | 5‘-TTTGGATCCTTAACTCAGTTCCTCCTGTACTTTTCT  TTTTG | Amplification of *ansR* |
| SM25 | 5‘-TTTAAATGTGAGCTTGCCCGCAAAAAAG | Construction of strain BP270 |
| SM26 | 5‘-TTTAGACAAGGGAACGATTATTATATTGGACA | Construction of strain BP270 |
| SM27 | 5‘-CCTATCACCTCAAATGGTTCGCTGCTTGAACTCCC  CCTAATTCGTCTTAAG | Construction of strain BP270 |
| SM28 | 5‘-CGAGCGCCTACGAGGAATTTGTATCGACAGATCA  AAAAGCGGCTGACAGAAAAG | Construction of strain BP270 |
| SM29 | 5‘-TTTTACGTAATTCTTGGGAACGGGGCT | Construction of strain BP270 |
| SM30 | 5‘-TTTGTGATGAATACCGGTTTGTCATAATGTTC | Construction of strain BP270 |
| SM36 | 5‘-TTTGAATTCTCATTAACTCAGTTCCTCCTGTACTTT  TCTTTTTGTG | Construction of pBP1111 |
| Tc fwd1 (kan) | 5‘-CAGCGAACCATTTGAGGTGATAGGGCTTATCAAC GTAGTAAGCGTGG | Amplification of the *tet* gene |
| Tc rev (kan) | 5‘-CGATACAAATTCCTCGTAGGCGCTCGGGAACTCT CTCCCAAAGTTGATCCC | Amplification of the *tet* gene |

^a^ Restriction sites are underlined.

**Table S3.** Plasmids

| **Plasmid** | **Description** | **Reference, construction** |
| --- | --- | --- |
| pAC7 | For the construction of translational *lacZ* fusions, integration into the *amyE* locus | Weinrauch et al., 1991 |
| pBP1110 | pAC7::*P_ansAB_* | This study |
| pBP1111 | pAC7:: *ansR-P_ansAB_* | This study |
| pDG647 | Template for the amplification of the *ermC* gene | Guérot-Fleury et al., 1995 |
| pDG1514 | Template for the amplification of the *tet* gene | Guérot-Fleury et al., 1995 |
| pDG1726 | Template for the amplification of the *spc* gene | Guérot-Fleury et al., 1995 |
| pGEM-cat | Template for the amplification of the *cat* gene | Laboratory collection |

**Supporting information references**

**Abe S, Takayama K, Kinoshita S** (1967) Taxonomical studies on glutamic acid-producing bacteria. *J Gen Appl Microbiol* 13: 279-301.

**Blasco B, Stenta M, Alonso-Sarduy L, Dietler G, Peraro MD, Cole ST, Pojer F.** 2011. Atypical DNA recognition mechanism used by the EspR virulence regulator of *Mycobacterium tuberculosis.* Mol Microbiol 82: 251-264.

**Guérout-Fleury AM, Shazand K, Frandsen N, Stragier P.** 1995. Antibiotic resistance cassettes for *Bacillus subtilis.* Gene 167: 335-336.

**Guo J, Coker AR, Wood SP, Cooper JB, Chohan SM, Rashi N, Akhtar M.** 2017. Structure and function of the thermostable L-asparaginase from *Thermococcus kodakarensis*. Acta Crystallogr D Struct Biol 73: 889-895.

**Meyer P, Evrin C, Briozzo P, Joly N, Barzu O, Gilles AM.** 2008. Structural and functional characterization of *Escherichia coli* UMP kinase in complex with its allosteric regulator GTP. J Biol Chem 283: 36011.

**Omasits U, Ahrens CH, Müller S, Wollscheid B.** 2014. Protter: interactive protein feature visualization and integration with experimental proteomic data. Bioinformatics 30: 884-886.

**Parris KD, Lin L, Tam A, Mathew R, Hixon J, Stahl M, Fritz CC, Seehra J, Somers WS.** 2000. Crystal structures of substrate binding to *Bacillus subtilis* holo-(acyl carrier protein) synthase reveal a novel trimeric arrangement of molecules resulting in three active sites. Structure 8: 883-895.

**Rehm N, Georgi T, Hiery E, Degner U, Schmiedl A, Burkovski A, Bott M** (2010) L-Glutamine as a nitrogen source for *Corynebacterium glutamicum*: derepression of the AmtR regulon and implications for nitrogen sensing. *Microbiology* 156: 3180-3193.

**Richts B, Hertel R, Potot S, Poehlein A, Daniel R, Schyns G, Prágai Z, Commichau FM** (2020) Complete genome sequence of the prototrophic *Bacillus subtilis* subsp. *subtilis* strain SP1. *Microbiol Resourc Announc* 9: e00825-20.

**Schwedt I, Schöne K, Eckert M, Pizzinato M, Winkler L, Knotkova B, Richts B, Hau JL, Steuber J, Mireles R, Noda-Garcia L, Fritz G, Mittelstädt C, Hertel R, Commichau FM** (2023) The low mutational flexibility of the EPSP synthase in *Bacillus subtilis* is due to a higher demand for shikimate pathway intermediates. *Environ Microbiol.* doi: 10.1111/1462-2920.16518.

**Waterhouse A, Bertoni M, Bienert S, Studer G, Tauriello G, Gumienny R** et al. 2018. SWISS-MODEL: homology modeling of protein structures and complexes. Nucleic Acids Res 46: W296-W303.

**Weinrauch Y, Msadek T, Kunst F, Dubnau D** (1991) Sequence and properties of *comQ*, a new competence regulatory gene of *Bacillus subtilis*. *J Bacteriol* 173: 5685-5693.

**Wicke D, Schulz LM, Lentes S, Scholz P, Poehlein A, Gibhardt J, Daniel R, Ischebeck T, Commichau FM** (2019) Identification of the first glyphosate transporter by genomic adaptation. *Environ Microbiol* 21: 1287-1305.
